# Analysis of the Mycosporine-Like Amino Acid (MAA) Pattern of the Salt Marsh Red Alga *Bostrychia scorpioides*

**DOI:** 10.3390/md19060321

**Published:** 2021-05-31

**Authors:** Maria Orfanoudaki, Anja Hartmann, Julia Mayr, Félix L. Figueroa, Julia Vega, John West, Ricardo Bermejo, Christine Maggs, Markus Ganzera

**Affiliations:** 1 Institute of Pharmacy/Pharmacognosy, University of Innsbruck, Innrain 80-82, 6020 Innsbruck, Austria; orfmaria@gmail.com (M.O.); Julia.mayr90@gmail.com (J.M.); markus.ganzera@uibk.ac.at (M.G.); 2 Experimental Centre Grice-Hutchinson, Institute of Blue Biotechnology and Development (IBYDA), University of Malaga, 29004 Malaga, Spain; felix_lopez@uma.es (F.L.F.); juliavega@uma.es (J.V.); 3 School of BioSciences, University of Melbourne, Parkville, VIC 3010, Australia; jwest@unimelb.edu.au; 4 Earth and Ocean Sciences, School of Natural Sciences and Ryan Institute, National University of Ireland, H91 TK33 Galway, Ireland; ricardo.bermejo@uca.es; 5 Medical Biology Centre, School of Biological Sciences, Queen’s University Belfast, Belfast BT22 1PF, UK; cmaggs@bournemouth.ac.uk

**Keywords:** *Bostrychia scorpioides*, mycosporine-like amino acids, bostrychines, method validation, HPLC-DAD

## Abstract

This study presents the validation of a high-performance liquid chromatography diode array detector (HPLC-DAD) method for the determination of different mycosporine-like amino acids (MAAs) in the red alga *Bostrychia scorpioides*. The investigated MAAs, named bostrychines, have only been found in this specific species so far. The developed HPLC-DAD method was successfully applied for the quantification of the major MAAs in *Bostrychia scorpioides* extracts, collected from four different countries in Europe showing only minor differences between the investigated samples. In the past, several *Bostrychia* spp. have been reported to include cryptic species, and in some cases such as *B. calliptera*, *B. simpliciuscula*, and *B. moritziana*, the polyphyly was supported by differences in their MAA composition. The uniformity in the MAA composition of the investigated *B. scorpioides* samples is in agreement with the reported monophyly of this *Bostrychia* sp.

## 1. Introduction

*Bostrychia scorpioides* (Hudson) Montagne ex Kützing is one of the most common red macroalgae growing in coastal salt marshes in Europe [1], and the only one found in Europe of approximately 40 *Bostrychia* species that are known globally [2]. It occurs on rocks, mud, and wood structures and partially as tufts on halophytes such as *Halimione portulacoides* in the upper and middle intertidal zones [3]. It can survive for a long period when exposed to air [1,4] by experiencing severe osmotic and desiccation stress. This taxon has been reported to produce different metabolites in comparison to other Rhodophyta, such as polyols, d-sorbitol, and d-dulcitol instead of the most common heterosides floridoside or digeneaside [5]. Moreover, unique mycosporine-like amino acids (MAAs) named bostrychines A–F occur instead of widely distributed derivatives such as porphyra-334 or shinorine [6].

MAAs are small, highly polar compounds that absorb UV radiation in the range of 310–365 nm [7]. They are composed of a cyclohexenone or a cyclohexenimine scaffold, carrying nitrogen or imino alcohol substituents [8]. They mainly play a photoprotective role, but they are also considered as osmoprotectants, scavengers of free oxygen radicals and protectants against desiccation and thermal stress [7,8,9]. According to several studies, the molecular diversity of *Bostrychia* species is greater than previously assumed, and this fact led to recircumscriptions of many species within the Bostrychieae [10]. It has been reported that some complexes include cryptic species and, in some cases, MAAs were used as chemotaxonomic markers for the identification of different chemotypes [11,12]. The unique bostrychines of *B. scorpioides* are completely absent in all other *Bostrychia* species and thus are very significant taxonomic characters. The numerous other chemicals of *Bostrychia* species have not yet been sufficiently assessed as taxonomic characters or, as yet, evaluated relative to the distinctly different habitats of these taxa. Careful investigations and publications are pending in contrast to reports on the chemodiversity and chemotaxonomy of other algal taxa, e.g., the presence of fatty acids in the family Selenastraceae (Chlorophyceae) [13], galactan structures in the family Gigartinaceae (Rhodophyceae) [14], acetogenins and linear or cyclized terpenoids in the family Cystoseiraceae (Phaeophyceae) [15], and bromophenols in *Leathesia* sp. (Chordariaceae, Ochrophyta) [16].

In this context, the main focus of the present study was to investigate the different MAA patterns in the salt marsh macroalga *B. scorpioides*. To investigate the phytochemical profile of different *B. scorpioides* samples and unravel possible geographic patterns, various isolates were collected from several coastal regions of Western Europe and analyzed with an HPLC-DAD method that was developed and validated for the quantification of the main MAAs in *Bostrychia scorpioides*. 

Validation was carried out following the ICH guidelines based on specificity, linearity, precision, and accuracy. Optimal chromatographic separation was achieved in 40 min on a YMS-Pack Pro C18 RS (150 × 4.6 mm, 3 µm) column by using water and methanol as mobile phase, modified with 0.9% (*v/v*) formic acid and 0.1% (*v/v*) acetic acid. The assay’s sensitivity, linearity (R^2^ ≥ 0.9996), precision (intraday precision ≤ 4.31%; interday precision ≤ 4.81%) and accuracy (recovery rates between 93.08% and 103.78%) were confirmed, rendering it suitable for the quantitative analysis of the main MAAs. Finally, practical applicability was proven by assaying different *Bostrychia scorpioides* extracts.

## 2. Results

### 2.1. Method Development

#### 2.1.1. Sample Preparation

Prior to sample analysis, optimum extraction and homogenization conditions were determined. A variety of extraction procedures (duration of the extraction or cycles of sonication) and solvents (water, and mixtures with different methanol content) were investigated. Ultrasound-assisted extraction of the plant material with 100% water as solvent was found to be most efficient, being the only procedure to enable the exhaustive extraction of all relevant compounds. Homogeneously powdered plant material required for reproducible measurements was only obtained after the use of a Mikro-Dismembrator (Sartorius, Göttingen, Germany). The efficiency of the applied extraction procedure was confirmed as follows: a sample was prepared as described in Section 4.4 and after the extraction of the plant material once more, the supernatant was analyzed by HPLC. As no quantifiable amounts of the marker compounds were found in this solution, the suitability and efficiency of the applied extraction procedure was proven.

#### 2.1.2. HPLC-DAD

For the development of an improved HPLC method, different stationary phases were examined, one from Phenomenex (Synergi MAX-RP 80A, 150 mm × 4.60 mm, 4 μm) and three from YMC (Triart C18, 150 mm × 3.00 mm, 3 μm; YMC-Pack ODS column, 250 mm × 4.60 mm, 5 μm; YMC-Pack Pro C-18 RS, 150 mm × 4.60 mm, 3 μm). The last column yielded the best results in terms of overall separation efficiency; thus, it was selected for further improvement.

Methanol and water have been commonly used as mobile phase for the HPLC separation of MAAs; however, even a very low percentage of methanol (1–2%) already resulted in poor retention of the analytes. Thus, purely aqueous conditions for the first 15 minutes of the method were selected. Adding methanol thereafter was advantageous for the elution and separation of the less polar MAAs. The addition of acidic modifiers like acetic acid and formic acid or buffers, such as ammonium formate, drastically improved the peak shape and resolution. Finally, the selected modifiers, 0.9% (*v/v*) formic acid and 0.1% (*v/v*) acetic acid, resulted in the overall best peak symmetry and resolution; therefore, they were utilized for further experiments. Also, temperature played an important role in the resolution of closely eluting MAAs. The separation of compounds **5** and **6** was improved at 20 °C while 30 °C was the best in order to resolve bostrychine B from other (currently unknown) constituents. Finally, as a compromise, column temperature was set to 25 °C. Flow rate proved to be very important as well, especially for the separation of bostrychine B from other unknown constituents. A lower flow rate of 0.55 mL min^−1^ was required to resolve them adequately. 

### 2.2. Method Validation

**Figure 1:**
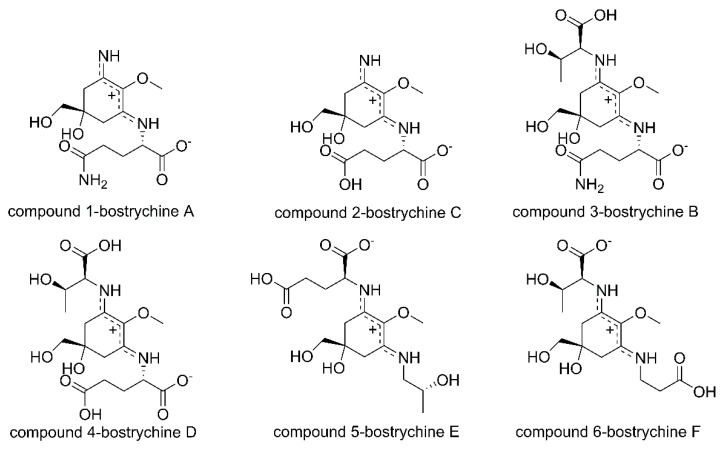
The structures of compounds **1**–**6**.

Several validation parameters indicate that the developed method is suitable for the quantitation of compounds **1**–**6** (Figure 1) in red algae. Calibration curves for all standards were constructed by plotting the peak areas against standard compound concentrations following a linear least-square fit regression model. Calibration data presented in Table 1 indicate linearity of the method in the tested range with a determination coefficient (R^2^) higher than 0.9996 in all cases. For bostrychine A, sufficient material with purity over 95% was not available; therefore it was quantified according to the calibration data of the structurally most similar bostrychine C. Limits of detection (LOD) and limits of quantification (LOQ) ranged from 0.04 μg·mL^−1^ to 0.22 μg·mL^−1^ and from 0.13 μg·mL^−1^ to 0.65 μg·mL^−1^, respectively (Table 1). Assay precision was assured by repeatedly extracting and analyzing sample 1. Intraday precision was found to be better than 4.31% and interday variation below 4.81% for all analytes (Table 1).

For determination of accuracy, only three marker compounds were utilized due to the limited number of standards available. Recovery rates were assessed at three different concentration levels (low, medium, and high) and ranged from 93.08% to 103.78% (Table 1).

### 2.3. Sample Analysis

**Figure 2:**
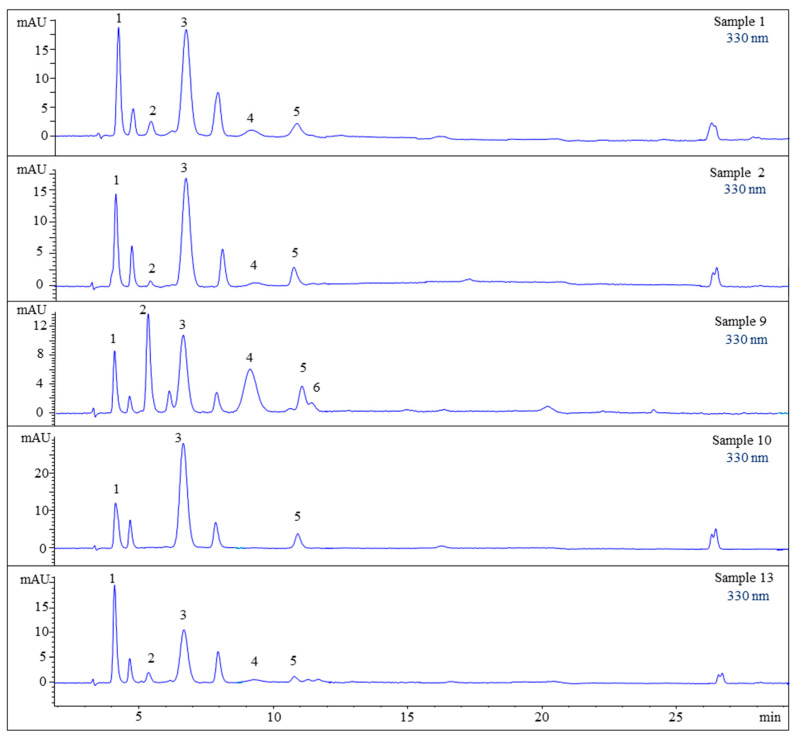
HPLC chromatograms of selected *B. scorpioides* samples. Assignment of the compounds is according to Figure 1.

Seventeen batches of *B. scorpioides* were analyzed with the developed HPLC-DAD method. The results shown in Table 2 and Figure 2 indicate that all six reference compounds could be identified in these samples (matching retention times and UV spectra), but only five could be quantified since bostrychine F was always present in concentrations lower than the LOQ. It has to be noted that only small quantitative differences were observed among the samples. Compounds **1** and **3** were present in all samples. Bostrychine D was quantifiable only in specimens from France, while bostrychine F could only be detected in samples from the same country (France). The only culture sample (sample 14) did not contain bostrychine E in contrast to all others which were collected in the field. Additionally, this sample yielded only compounds **2** and **3**, but at higher concentrations compared to field samples.

## 3. Discussion

In the present study, the MAA pattern of the red macroalga *B. scorpioides* was investigated. The respective compounds have already been reported previously by the same authors, and in the current work an HPLC-DAD method was developed and validated for the quantification of the major MAAs in *Bostrychia scorpioides* extracts for the first time. Satisfactory validation data for all tested parameters, including sensitivity, linearity, precision, and accuracy, as well as practical applicability, render the developed method a useful and reliable tool for the determination of MAAs in *B. scorpioides*.

In the past, a few studies focused on the quantitative and qualitative analysis of mycosporine-like amino acids and the most common technique is reversed-phase HPLC using C-8 and C-18 stationary phases [17,18,19,20]. Capillary electrophoresis (CE) and hydrophilic interaction liquid chromatography (HILIC) have also been described [21,22]. The major disadvantage of most studies is the low number of examined analytes with the exception of the study presented by Carreto et. al (2005) [23], who used two C18 columns connected in series for the analysis of more than 20 MAAs [17,18,19,20,21,22]. In general, the main problem in MAA analysis is the low retention of these metabolites on the column material, which results in low resolution and selectivity, especially when a large number of MAAs are analyzed. A prior study by some of the co-authors led to the method development and validation of an HPLC method that allowed the quantification of the 12 most common MAAs of Rhodophyta in 35 min using a YMC-Pack ODS column [12]. However, bostrychines of *B. scorpioides* extracts were not properly resolved with this method. As a consequence, the development of a new HPLC method was necessary due to the significance of these metabolites as chemotaxonomic markers.

Compared to all other *Bostrychia* spp., which are completely absent in Europe, *B. scorpioides* occupies intertidal habitats of the European Atlantic coast, and shows a unique MAA composition, producing none of the common MAAs such as porphyra-334, palythine-threonine, aplysiapalythine A, asterina-330 or shinorine, which are present in most of the other species [6,12,24]. Thus, bostrychines are very significant chemotaxonomic characters allowing the discrimination of this species. MAAs have been used in the past as biochemical indicators of taxonomy, e.g., for the discrimination of *Grateloupia* sp. of the Iberian Peninsula [25], for chemotaxonomic studies in the *Prasiola*-clade (Trebouxiophyceae, Chlorophyta) [26], or for the identification of cryptic species in the *Bostrychia* genus [11,12]. There are two proposed pathways for MAA biosynthesis, both identifying 4-deoxygadusol (4-DG) as the precursor of MAAs: the shikimate pathway and the pentose phosphate pathway [27]. Bostrychines contain the cyclohexenimine scaffold, which is common among MAAs produced by other red algae; however, the combination of amino acids in the side chains of bostrychines, mainly glutamine, glutamic acid or threonine, is not found in other MAAs. The reason for the presence of these specific amino acids may be because of different roles of these MAAs in the alga [7] or variations in their biosynthetic pathways, especially in late biosynthesis steps when amino-moieties are added to the core [28].

Even if the quantitative results were similar, small differences could be observed among the *B. scorpioides* samples analyzed. Compounds **1** and **3** were always the main MAAs; however, samples from France seemed to produce a higher variety of compounds compared to specimens from the UK, Ireland, and Spain. Moreover, the only sample provided from culture contained the highest concentration of bostrychine C as well as the second-highest concentration of bostrychine A, but it did not produce bostrychine E, which was found in all the field samples. This may be explained by the absence of desiccation stress, lower UV exposure due to the use of LEDs during cultivation, or differences in the concentration of nutrients in the culture medium.

In contrast to other *Bostrychia* spp. such as *B. calliptera* and *B. simpliciuscula*, which are reported to be polyphyletic and show distinct differences in their MAA composition [11,12], no qualitative difference was found in the MAA pattern of the monophyletic species *B. scorpioides*.

## 4. Materials and Methods

### 4.1. Plant Material

Some of the authors (Maria Orfanoudaki, Félix L. Figueroa, Julia Vega, John West, Ricardo Bermejo, Christine Maggs) collected and morphologically identified the isolates, using their taxonomic expert knowledge in conjunction with standard identification keys [2,22]. Voucher samples of specimens 1–13 and 15–17 are deposited at the Department of Pharmacognosy, University of Innsbruck, Austria, and sample 14 at the School of BioSciences, University of Melbourne, Victoria, Australia. Further information regarding collection date and place are summarized in Table S1.

### 4.2. Chemicals 

Methanol had pro-analysis (p.a) quality and was purchased from Merck (Darmstadt, Germany). Formic acid and acetic acid were obtained from VWR International (Vienna, Austria). A Sartorius Arium^®^ 611 UV (Sartorius, Göttingen, Germany) purification system was used for the production of ultrapure water. 

### 4.3. MAA Isolation 

The isolation of the standard compounds was described previously [6]. Assignment of the compounds is as follows: bostrychine A (compound **1**), bostrychine B (compound **3**), bostrychine C (compound **2**), bostrychine D (compound **4**), bostrychine E (compound **5**), and bostrychine F (compound **6**). Original NMR and UV spectra are shown in the supplementary materials (Figures S1–S39). The identity of the standards was determined by NMR, and MS, and their purity was found to be higher than 95%. NMR experiments were conducted on a Bruker Avance II 600 spectrometer (Bruker, Karlsruhe, Germany) operating at 600.19 (^1^H) and 150.91 MHz (^13^C). The isolated compounds were dissolved in deuterated water from Euriso-Top (Saint Aubin, France) using tetramethylsilane (TMS) as an internal standard.

### 4.4. HPLC Sample Preparation 

For the preparation of each sample, 100 mg of *B. scorpioides* plant material was frozen in liquid nitrogen and homogenized using a Mikro-Dismembrator (Sartorius, Göttingen, Germany). The homogenization procedure was done separately for each of the different experiments (quantitation, precision, and accuracy). For extraction, 7.0 mL of water was added to the plant material, the sample was mixed on a Vortex mixer (VWR, Vienna, Austria) and afterwards extracted in an ultrasonic bath (15 min at room temperature). After centrifugation at 2000× *g* for 2 min the supernatant was placed in a 25 mL volumetric flask. This procedure was repeated twice and subsequently the flask was filled up to the final volume with the extraction solvent. Finally, the sample solution was filtered through 0.45 µm Phenex-RC 4 mm syringe filters (Phenomenex, Torrance, CA, USA).

### 4.5. HPLC-DAD Analysis

HPLC analyses were performed on an Agilent 1100 series HPLC instrument, equipped with a quaternary pump, an autosampler, a column oven, and a photodiode array detector (Agilent, Waldbronn, Germany). Optimum separation was achieved on a YMS-Pack Pro C18 RS (150 × 4.6 mm, 3 µm) column from YMC, guarded with an in-line filter and using a mobile phase consisting of 0.9% (*v/v*) formic acid and 0.1% (*v/v*) acetic acid in water (A) and methanol (B). The applied gradient was as follows: 0% B at 0 min, 0% B at 15 min, 10% B at 23 min, 15% B at 30 min, and 98% B at 35 min, and held at this composition for 5 min (total runtime of 40 min); for the next 15 min the column was equilibrated under initial conditions. Flow rate, temperature, and injection volume were adjusted to 0.55 mL· min^−1^, 25 °C, and 4.0 μL. The detection wavelength was set to 330 nm. 

### 4.6. Calibration and Method Validation

The developed HPLC method was validated as per ICH guidelines. All validation results are summarized in Table 1.

#### 4.6.1. Linearity, Limit of Detection (LOD), and Limit of Quantification (LOQ) 

Standard stock solutions were prepared by separately weighting and dissolving compounds **2**–**6** in 100% water. At least nine calibration levels were prepared from each stock solution by dilution with pure water, and each level was assayed in triplicate (calibration data are presented in Table 1). Plotting the peak areas versus the concentrations of each analyte resulted in the generation of calibration curves. The regression parameters (intercept, slope, and determination coefficient (R²)) were calculated by linear regression analysis using Microsoft Excel. The LOD and LOQ values for each analyte were calculated utilizing only the lowest four dilution levels. LOD is defined as 3.3 times the residual standard deviation of the regression line divided by the slope of the calibration curve, whereas the LOQ corresponds to 10 times the residual standard deviation divided by the slope. 

#### 4.6.2. Precision and Accuracy

Analysis of three individually homogenized and prepared extracts of sample 1 on three consecutive days in triplicate assured the precision of this method. Intra- and interday assay variance was expressed as the relative standard deviation (RSD) of the replicate quantitative measurement of compounds **2**–**6**. Accuracy was determined by recovery experiments at three different concentration levels (low, medium, high) for analytes **2**, **3** and **6** since sufficient amounts of analytes **4** and **5** were not available for spiking experiments. For this purpose, individually homogenized portions of sample 1 were spiked with known amounts of analytes **2**, **3** and **6** prior to sample workup. All samples were prepared in triplicate.

## Figures and Tables

**Table 1:** Validation parameters of the HPLC method.

**Calibration Data**						
**Substance**	**Regression Equation ^a^**	**Coefficient of Determination**		**Linearity ^b^** **(μg··mL^−1^)**	**LOD ^c^** **(μg·mL^−1^)**	**LOQ ^d^** **(μg·mL^−1^)**
bostrychine C	y = 22248x + 1.4832	R² = 0.9996		0.06–105.20	0.07	0.23
bostrychine B	y = 29657x + 2.0058	R² = 0.9999		0.11–71.40	0.04	0.13
bostrychine D	y = 35033x + 8.1483	R² = 0.9997		0.26–83.00	0.13	0.40
bostrychine E	y = 42335x + 5.4806	R² = 0.9999		0.04–69.00	0.10	0.29
bbostrychine F	y = 27415x + 3.7719	R² = 0.9999		0.10–63.60	0.22	0.65
**Accuracy and Precision**						
**Precision**			**Accuracy ^g^**			
**Substance**	**Intraday ^e^**	**Interday ^f^**	**Substance**	**Low**	**Medium**	**High**
bostrychine A	2.45	2.91	bostrychine C	95.15 ± 1.03	103.06 ± 0.18	95.39 ± 0.11
bostrychine C	3.49	4.44	bostrychine B	95.53 ± 0.52	95.15 ± 2.99	97.75 ± 0.16
bostrychine B	1.97	2.06	bostrychine F	102.77 ± 0.40	95.15 ± 0.72	103.78 ± 0.85
bostrychine D	3.78	3.60				
bostrychine E	4.31	4.81				

^a^ x: concentration in mg·mL^−1^, y: AUC; ^b^ linear range; ^c^ LOD: limit of detection; ^d^ LOQ: limit of Quantification; ^e^ relative standard deviation within one day based on the peak area in percent; ^f^ Relative standard deviation within three days based on peak area in percent; ^g^ recovery rates in percent (mean ± RSD).

**Table 2:** Quantitative HPLC-DAD results for compounds **1**–**6** in *B. scorpioides* samples; all values expressed as mg per g of dry material; free spaces correspond to non-detected compounds; bostrychine A (compound **1**), bostrychine B (compound **3**), bostrychine C (compound **2**), bostrychine D (compound **4**), bostrychine E (compound **5**), and bostrychine F (compound **6**).

Sample	Bostrychine A (mg·g^−1^) (Srel% ^a^)	Bostrychine C (mg·g^−1^) (Srel%)	Bostrychine B (mg·g^−1^) (Srel%)	Bostrychine D (mg·g^−1^) (Srel%)	Bostrychine E (mg·g^−1^) (Srel%)	Bostrychine F (mg·g^−1^) (Srel%)
1	2.04 (2.91)	0.40 (4.43)	3.30 (2.06)	0.20 (3.60)	0.31 (4.81)	
2	1.8 (1.10)	0.14 (2.80)	3.15 (0.75)	<LOQ ^b^	0.28 (4.98)	
3	3.35 (0.54)	<LOQ ^b^	6.30 (0.66)		0.47 (4.96)	
4	3.16 (0.78)	0.22 (3.33)	4.03 (0.62)	0.16 (4.47)	0.21 (4.52)	<LOQ ^b^
5	2.49 (1.63)	0.25 (1.83)	3.04 (2.80)	0.18 (2.01)	0.21 (2.35)	<LOQ ^b^
6	2.84 (1.28)	0.07 (2.59)	3.85 (0.20)	0.02 (3.17)	0.24 (4.24)	
7	2.17 (0.36)	0.16 (4.38)	3.49 (0.49)	0.11 (3.62)	0.17 (2.10)	
8	1.87 (0.29)	<LOQ	2.35 (0.42)		0.14 (2.15)	
9	0.88 (2.31)	1.76 (3.00)	1.10 (3.37)	0.73 (4.47)	0.33 (4.26)	<LOQ ^b^
10	1.63 (0.50)		3.14 (0.41)		0.28 (5.16)	
11	1.46 (1.66)	0.11 (4.17)	1.95 (2.50)		0.16 (4.42)	
12	8.05 (0.47)	<LOQ ^b^	2.04 (0.26)		0.11 (4.86)	
13	1.96 (0.96)	0.30 (3.95)	1.14 (1.03)	<LOQ ^b^	0.08 (4.90)	
14	4.42 (1.73)		6.44 (0.74)			
15	1.53 (1.31)	0.14 (2.41)	1.11 (0.65)	<LOQ ^b^	0.09 (3.44)	
16	2.49 (0.61)		5.66 (0.27)		0.18 (3.40)	
17	1.42 (1.29)		3.94 (0.52)		0.39 (1.73)	

^a^ Relative standard deviation; ^b^ Concentration was higher than the limit of detection but lower than the limit of quantification.

## Data Availability

Data is contained within the article or supplementary material.

## Supplementary Materials

The following is available online at https://www.mdpi.com/article/10.3390/md19060321/s1: Table S1: Overview of the investigated *B. scorpioides* samples, including collection sites and dates, and NMR and UV spectra of the bostrychines. Figure S1. ^1^H NMR spectrum of bostrychine A in D_2_O at 400 MHz. Figure S2. COSY spectrum of bostrychine A in D_2_O at 400 MHz. Figure S3. HSQC spectrum of bostrychine A in D_2_O at 400 MHz. Figure S4. HMBC spectrum of bostrychine A in D_2_O at 400 MHz. Figure S5. ^13^C NMR spectrum of bostrychine A in D_2_O at 100 MHz. Figure S6. NOESY spectrum of bostrychine A in D_2_O at 400 MHz. Figure S7. UV spectrum of bostrychine A. Figure S8. ^1^H NMR spectrum of bostrychine B in D_2_O at 600 MHz. Figure S9. COSY spectrum of bostrychine B in D_2_O at 600 MHz. Figure S10. HSQC spectrum of bostrychine B in D_2_O at 600 MHz. Figure S11. HMBC spectrum of bostrychine B in D_2_O at 600 MHz. Figure S12. ^13^C NMR spectrum of bostrychine B in D_2_O at 150 MHz. Figure S13. UV spectrum of bostrychine B. Figure S14. ^1^H NMR spectrum of bostrychine C in D_2_O at 600 MHz. Figure S15. COSY spectrum of bostrychine C in D_2_O at 600 MHz. Figure S16. HSQC spectrum of bostrychine C in D_2_O at 600 MHz. Figure S17. HMBC spectrum of bostrychine C in D_2_O at 600 MHz. Figure S18. ^13^C NMR spectrum of bostrychine C in D_2_O at 150 MHz. Figure S19. UV spectrum of bostrychine C. Figure S20. ^1^H NMR spectrum of bostrychine D in D_2_O at 600 MHz. Figure S21. COSY spectrum of bostrychine D in D_2_O at 600 MHz. Figure S22. HSQC spectrum of bostrychine D in D_2_O at 600 MHz. Figure S23. HMBC spectrum of bostrychine D in D_2_O at 600 MHz. Figure S24. ^13^C NMR spectrum of bostrychine D in D_2_O at 150 MHz. Figure S25. UV spectrum of bostrychine D. Figure S26. ^1^H NMR spectrum of bostrychine E in D_2_O at 600 MHz. Figure S27. COSY spectrum of bostrychine E in D_2_O at 600 MHz. Figure S28. ^13^C NMR spectrum of bostrychine E in D_2_O at 150 MHz. Figure S29. HSQC spectrum of bostrychine E in D_2_O at 600 MHz. Figure S30. HMBC spectrum of bostrychine E in D_2_O at 600 MHz. Figure S31. NOESY spectrum of bostrychine E in D_2_O at 600 MHz. Figure S32. UV spectrum of bostrychine E. Figure S33.^1^H NMR spectrum of bostrychine F in D_2_O at 600 MHz. Figure S34. COSY spectrum of bostrychine F in D_2_O at 600 MHz. Figure S35. HSQC spectrum of bostrychine F in D_2_O at 600 MHz. Figure S36. HMBC spectrum of bostrychine F in D_2_O at 600 MHz. Figure S37. ^13^C NMR spectrum of bostrychine F in D_2_O at 150 MHz. Figure S38. NOESY spectrum of bostrychine F in D_2_O at 600 MHz. Figure S39. UV spectrum of bostrychine F.
