# Supplementary material for: Analysis of the Mycosporine-Like Amino Acid (MAA) Pattern of the Salt Marsh Red Alga Bostrychia scorpioides
Source: Mar Drugs. 2021 May 31;19(6):321. doi: 10.3390/md19060321 (PMC8229764; doi:10.3390/md19060321)
Supplement: Supplementary file 1 [file marinedrugs-19-00321-s001.zip › marinedrugs-1185932-supplementary.pdf]

Analysisof the Mycosporine-Like Amino Acid (MAA) Pattern of theSalt Marsh Red Alga *Bostrychia scorpioides*

Maria Orfanoudaki[a], Anja Hartmann[a,*], Julia Mayr, Félix L. Figueroa [b], Julia Vega [b], John West[c], Ricardo Bermejo[d], Christine Maggs, and Markus Ganzera[a]

[a] Institute of Pharmacy, Pharmacognosy, University of Innsbruck, Innrain 80-82, Innsbruck 6020, Austria; orfmaria@gmail.com (M.O.);anja.hartmann@uibk.ac.at (A.H.); julia_mayr1@gmx.at (J. M.); markus.ganzera@uibk.ac.at (M.G.)

[b]University of Malaga, Institute of Blue Biotechnology and Development (IBYDA), Experimental Centre Grice-Hutchinson, Lomas de San Julian, 29004-Malaga, Spain; felix_lopez@uma.es (F.L.F.); juliavega@uma.es (J.V.)

[c] School of BioSciences, University of Melbourne, Parkville, 3010 Victoria, Australia; jwest@unimelb.edu.au (J. W.)

[d] Earth and Ocean Sciences, School of Natural Sciences and Ryan Institute, National University of Ireland, Galway, H91 TK33, Ireland; ricardo.bermejo@uca.es (R. B.)

[e] Christine Maggs, School of Biological Sciences and Queen's University Marine Laboratory Portaferry, Queen's University Belfast, Northern Ireland, BT22 1PF, UK

[*]Correspondence: anja.hartmann@uibk.ac.at (A.H.); Tel.: +43 512 507-58430

Contents

Table S1. Overview of the investigated B. scorpioides samples, their collection sites and dates. 4

Figure S1. ^{1}H NMR spectrum of bostrychine A in D_2O at 400 MHz 5

Figure S2. COSY spectrum of bostrychine A in D_2O at 400 MHz.. 5

Figure S3. HSQC spectrum of bostrychine A in D_2O at 400 MHz... 6

Figure S4. HMBC spectrum of bostrychine A in D_2O at 400 MHz.. 6

Figure S5. ^{13}C NMR spectrum of bostrychine A in D_2O at 100 MHz 7

Figure S6. NOESY spectrum of bostrychine A in D_2O at 400 MHz 7

Figure S7. UV spectrum of bostrychine A.. 8

Figure S8. ^{1}H NMR spectrum of bostrychine B in D_2O at 600 MHz 8

Figure S9. COSY spectrum of bostrychine Bin D_2O at 600 MHz 9

Figure S10. HSQC spectrum of bostrychine B in D_2O at 600 MHz 9

Figure S11. HMBC spectrum of bostrychine B in D_2O at 600 MHz... 10

Figure S12.^{13}C NMR spectrum of bostrychine B in D_2O at 150 MHz 10

Figure S13.UV spectrum of bostrychine B 11

Figure S14.^{1}H NMR spectrum of bostrychine C in D_2O at 600 MHz 11

Figure S15. COSY spectrum of bostrychine C in D_2O at 600 MHz 12

Figure S16. HSQC spectrum of bostrychine C in D_2O at 600 MHz 12

Figure S17. HMBC spectrum of bostrychine C in D_2O at 600 MHz... 13

Figure S18.^{13}C NMR spectrum of bostrychine C in D_2O at 150 MHz 13

Figure S19.UV spectrum of bostrychine C 14

Figure S20.^{1}H NMR spectrum of bostrychine D in D_2O at 600 MHz 14

Figure S21. COSY spectrum of bostrychine D in D_2O at 600 MHz.. 15

Figure S22. HSQC spectrum of bostrychine D in D_2O at 600 MHz.. 15

Figure S23. HMBC spectrum of bostrychine D in D_2O at 600 MHz... 16

Figure S24.^{13}C NMR spectrum of bostrychine D in D_2O at 150 MHz 16

Figure S25.UV spectrum of bostrychine D... 17

Figure S26.^{1}H NMR spectrum of bostrychine E in D_2O at 600 MHz... 17

Figure S27. COSY spectrum of bostrychine E in D_2O at 600 MHz 18

Figure S28.^{13}C NMR spectrum of bostrychine E in D_2O at 150 MHz 18

Figure S29. HSQC spectrum of bostrychine E in D_2O at 600 MHz 19

Figure S30. HMBC spectrum of bostrychine E in D_2O at 600 MHz 19

Figure S31. NOESY spectrum of bostrychine E in D_2O at 600 MHz 20
Figure S32.UV spectrum of bostrychine E 20
Figure S33.^{1}H NMR spectrum of bostrychine F in D_2O at 600 MHz 21
Figure S34. COSY spectrum of bostrychine F in D_2O at 600 MHz 21
Figure S35. HSQCspectrum of bostrychine F in D_2O at 600 MHz 22
Figure S36. HMBC spectrum of bostrychine F in D_2O at 600 MHz 22
Figure S37^{13}C NMR spectrum of bostrychine F in D_2O at 150 MHz 23
Figure S38. NOESY spectrum of bostrychine F in D_2O at 600 MHz 23
Figure S39.UV spectrum of bostrychine F 24

Table S1. Overview of the investigated B. scorpioides samples, their collection sites and dates.

Sample	Collection Place	Collection Date	Origin	Longitude	Latitude
1	Plouescat, France	10/06/2019	Field	-4.222010	48.648812
2	Sene, France	31/05/2019	Field	-2.743194	47.623058
3	Hillion, France	03/6/2019	Field	-2.676122	48.514435
4	Saint Colombier, France	31/05/2019	Field	-2.734889	47.552583
5	Saint Armel, Chenal de Saint Leonard, France	31/05/2019	Field	-2.713022	47.589354
6	Promenade des Rosvelec, Vannes, France	30/05/2019	Field	-2.748475	47.6209582
7	Saint Armel, Passage A lile de Tascon, France	31/05/2019	Field	-2.726754	47.569157
8	Kerbourbon, Vannes, France	30/05/2019	Field	-2.754591	47.6344224
9	Plouescat, France	14/06/2018	Field	-4.222010	48.648812
10	Miño, Galicia, Spain	October 2019	Field	- 8.206211	43.352718
11	Puente Lavaera, San Fernando, Cádiz, Spain	14/07/2019	Field	6.191112	36.4575345
12	Salt marsh of Palmones, Spain	12/07/2019	Field	-5.439694	36.173694
13	Burnham-on-Crouch, Essex, United Kingdom	01/09/2019	Field	0.843817	51.623467
14	Finavarra Demesne, Co. Clare, Ireland	03/09/2015	Culture	-9.091785	53.147615
15	Lettermore, Ireland	October 2019	Field	- 9.711216	53.2983547
16	Tolka, Ireland	October 2019	Field	- 6.157918	53.37177582
17	Argideen-Timoleague, Ireland	October 2019	Field	- 8.764898	51.641662

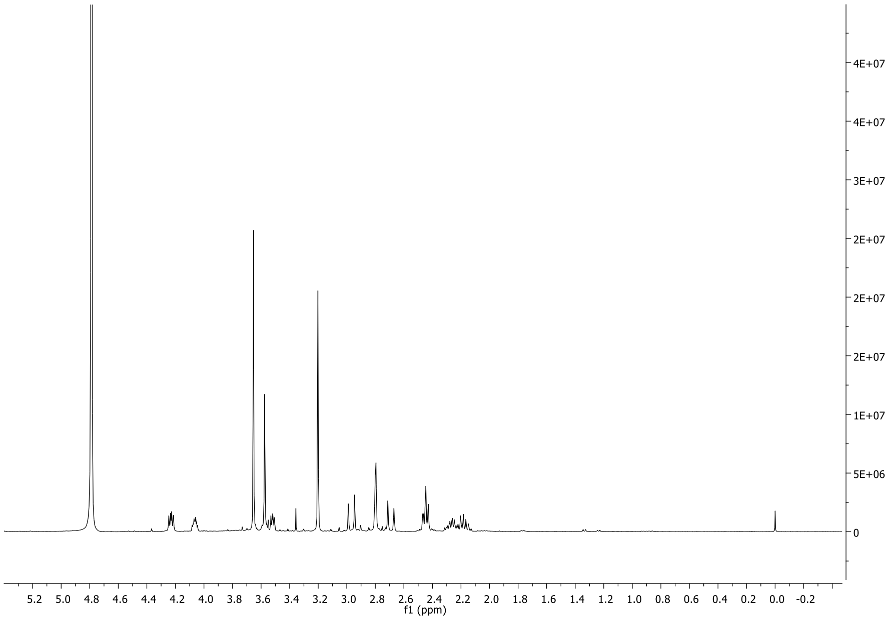

Figure S1. ^{1}H NMR spectrum of bostrychine A in D_2O at 400 MHz

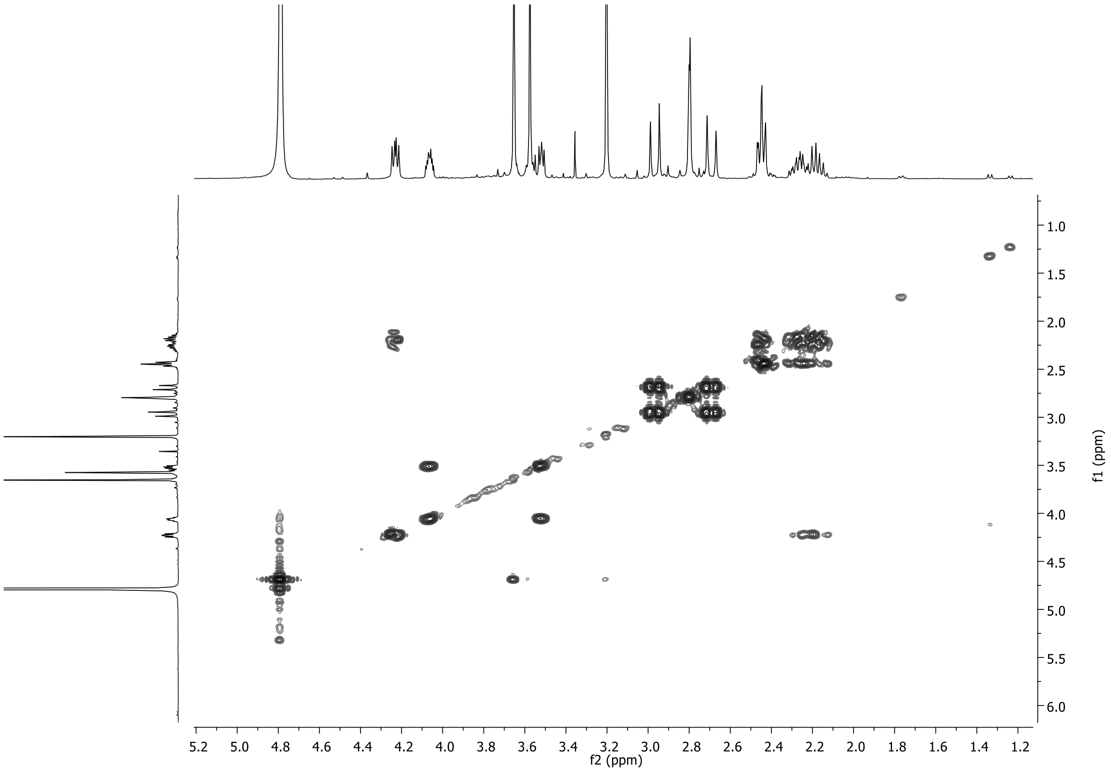

Figure S2. COSY spectrum of bostrychine A in D_2O at 400 MHz

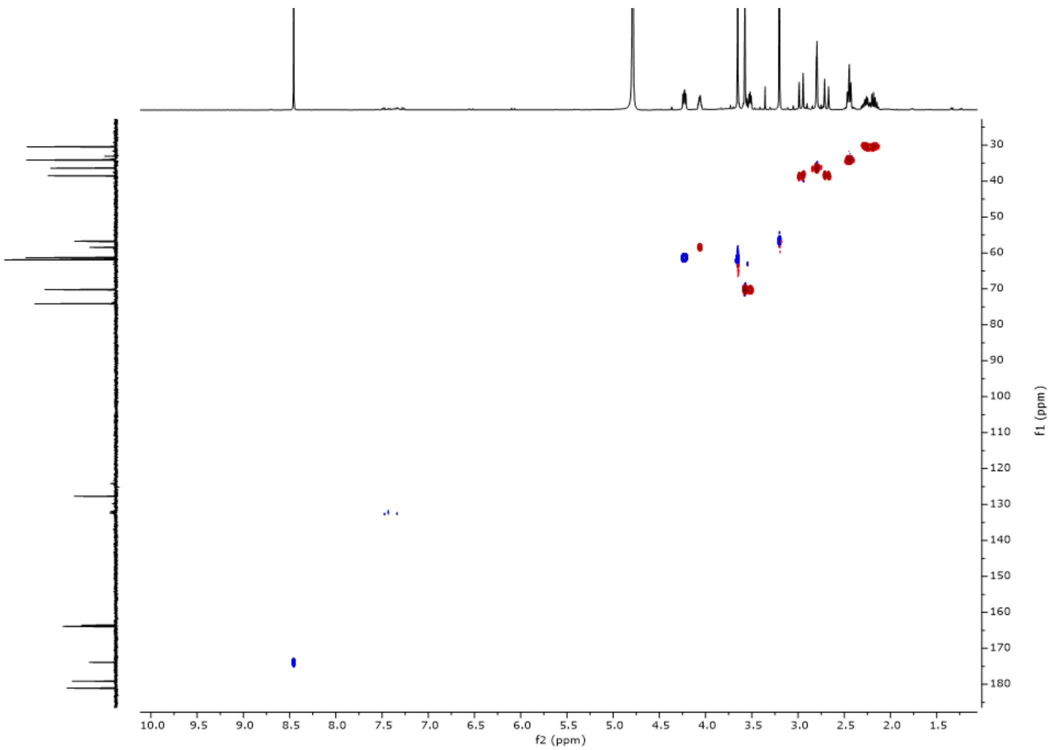

Figure S3. HSQC spectrum of bostrychine A in D_2O at 400 MHz

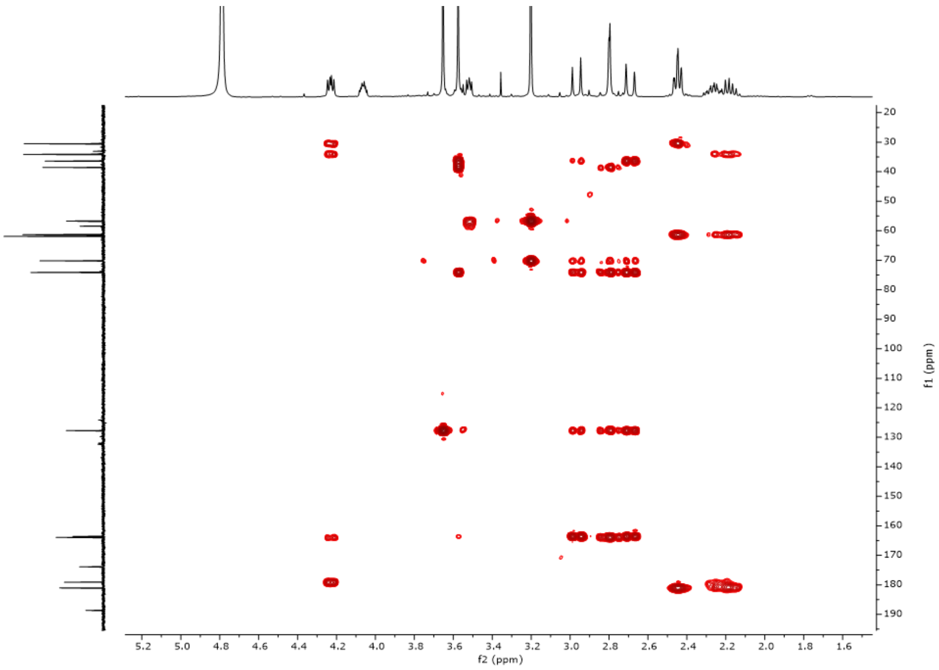

Figure S4. HMBC spectrum of bostrychine A in D_2O at 400 MHz

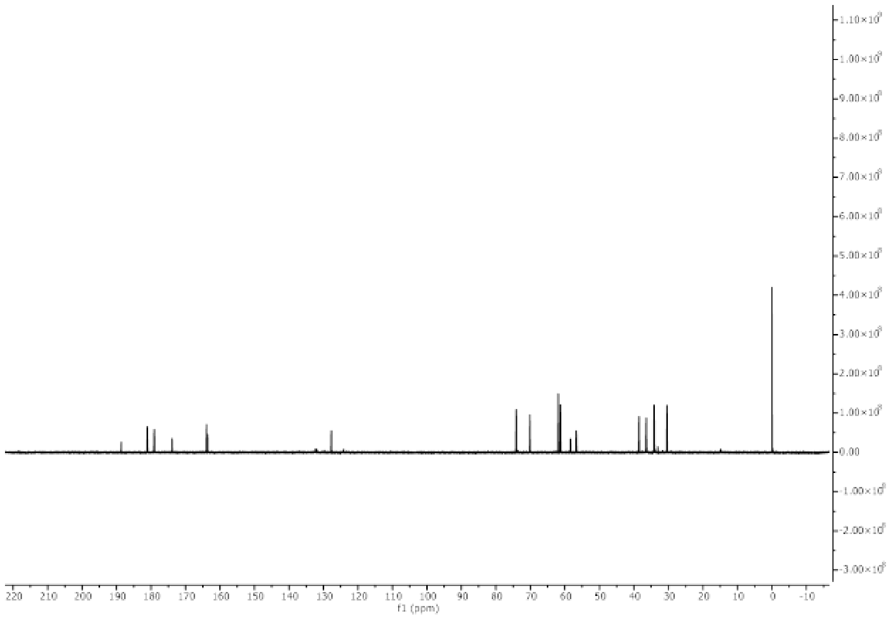

Figure S5. ^{13}C NMR spectrum of bostrychine A in D_2O at 100 MHz

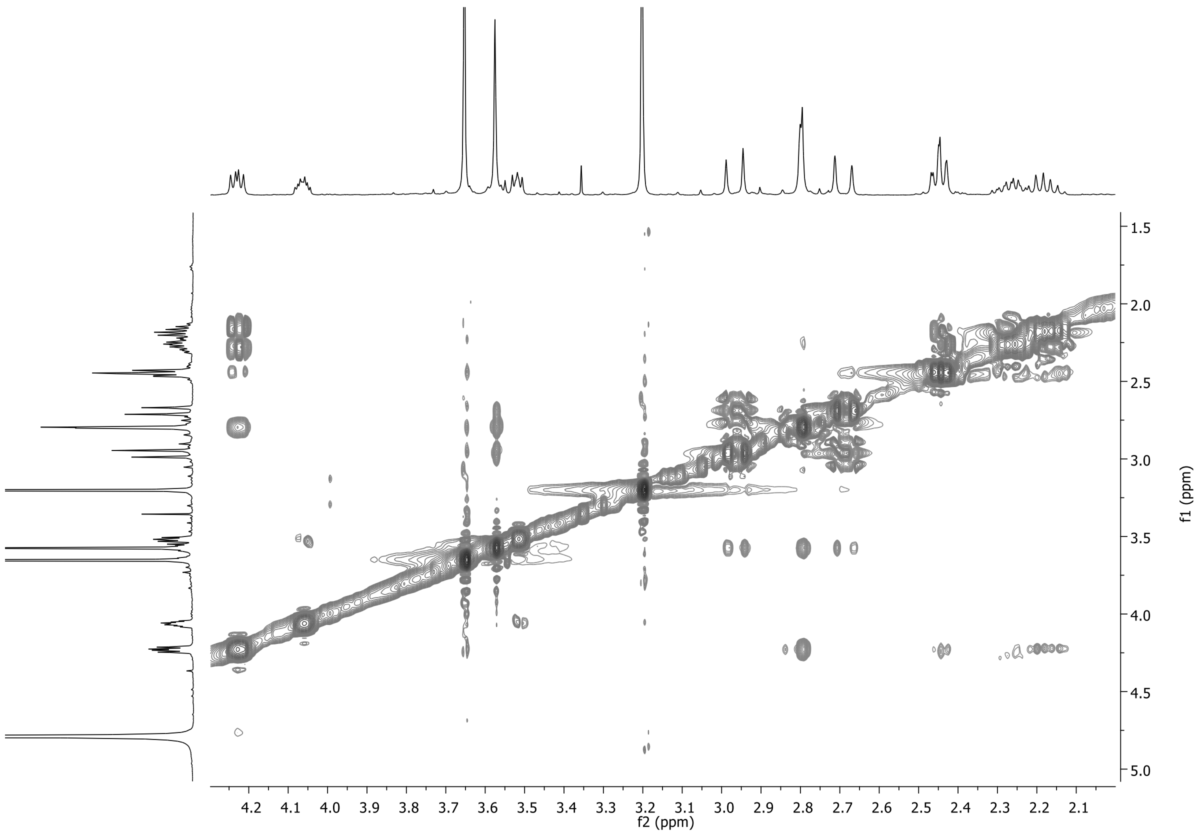

Figure S6. NOESY spectrum of bostrychine A in D_2O at 400 MHz

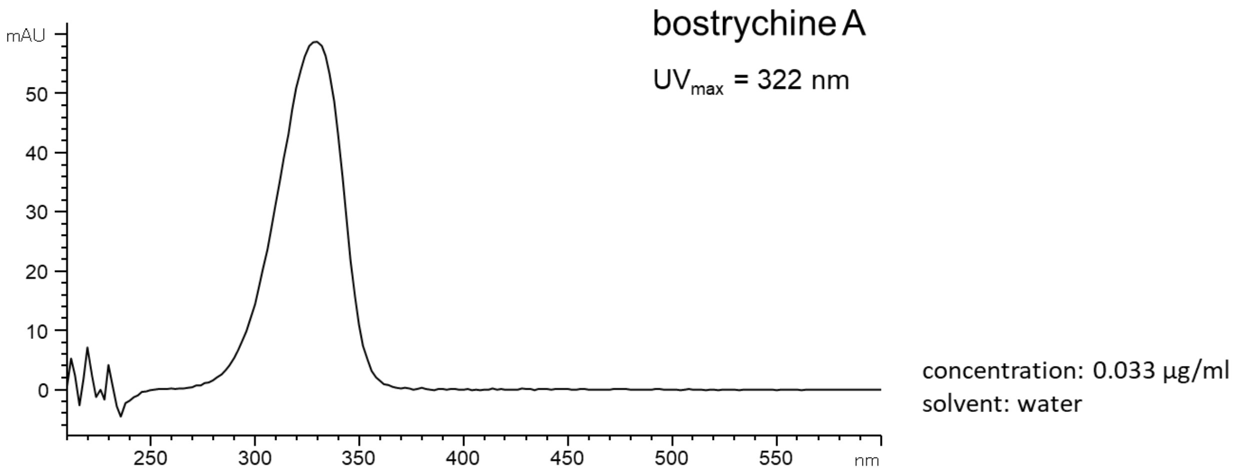

Figure S7. UV spectrum of bostrychine A

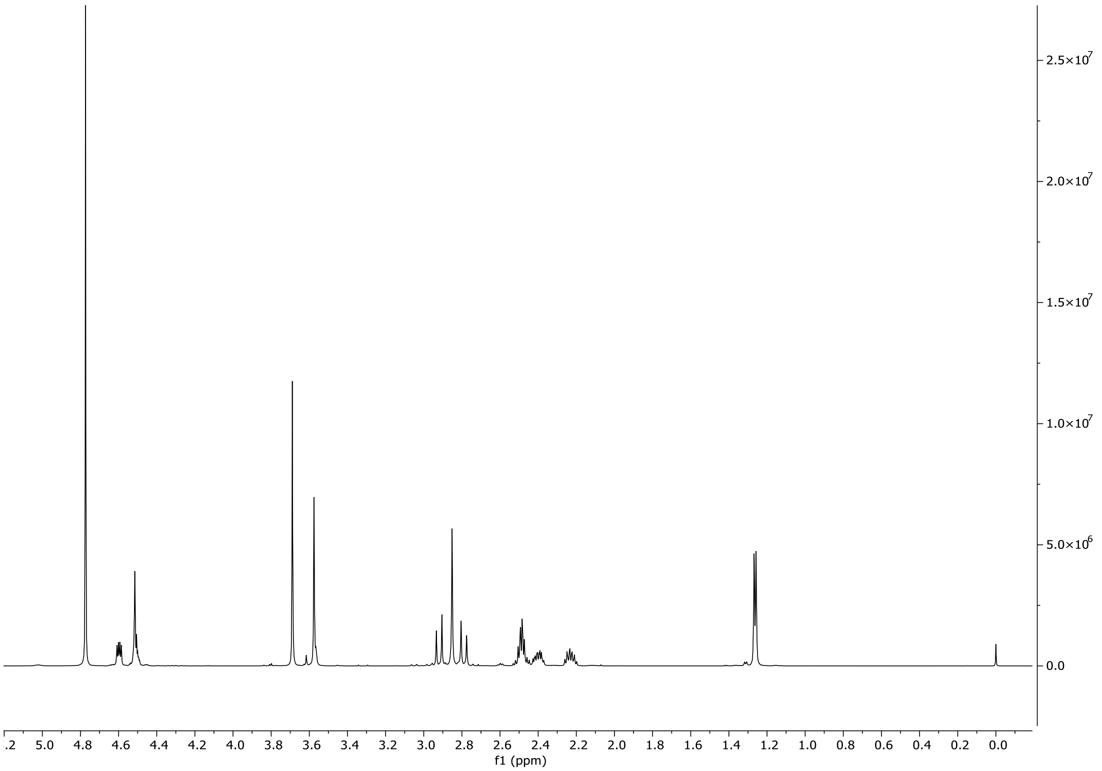

Figure S8. ^{1}H NMR spectrum of bostrychine B in D_2O at 600 MHz

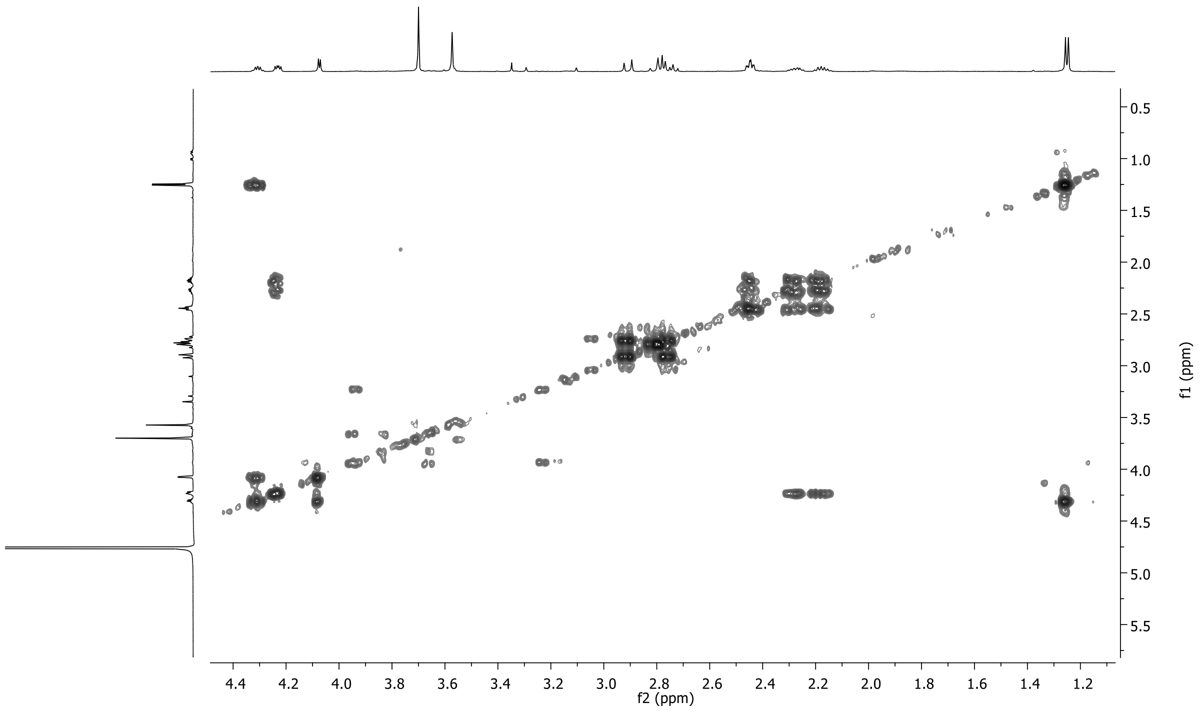

Figure S9. COSY spectrum of bostrychine B in D_2O at 600 MHz

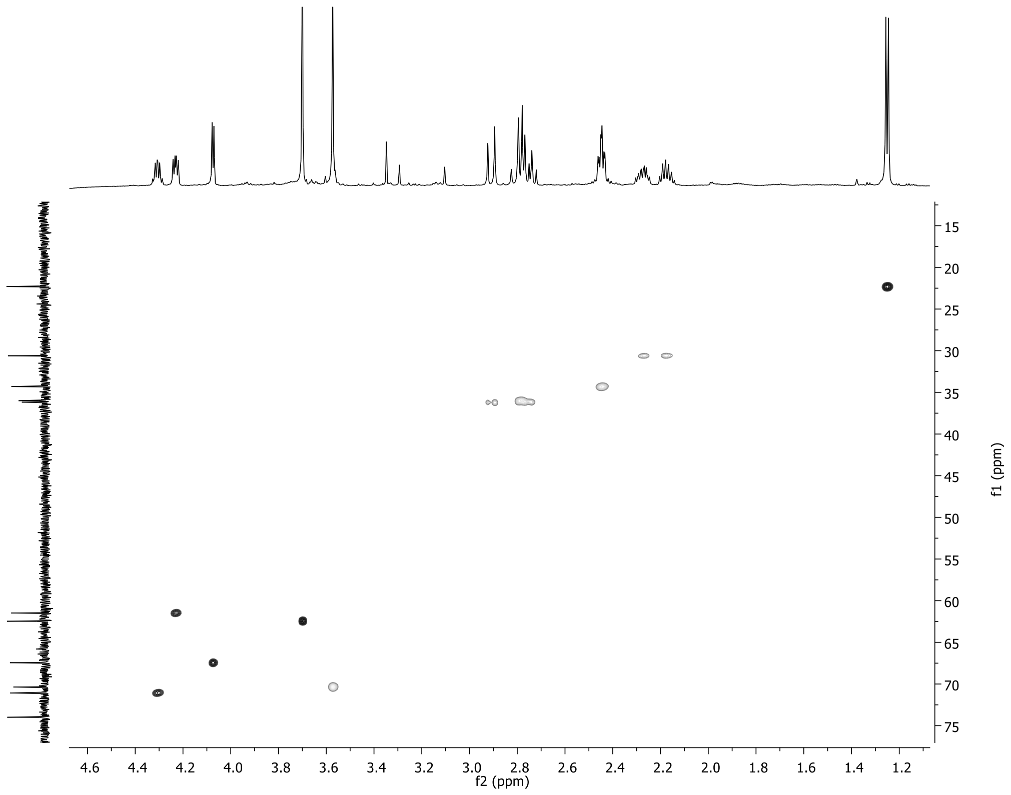

Figure S10. HSQC spectrum of bostrychine B in D_2O at 600 MHz

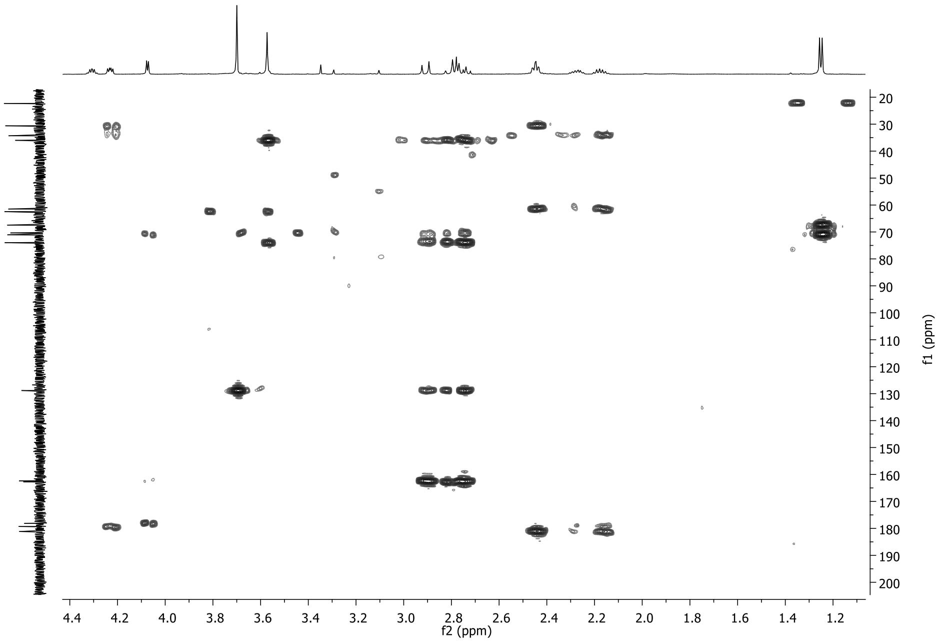

Figure S11. HMBC spectrum of bostrychine B in D_2O at 600 MHz

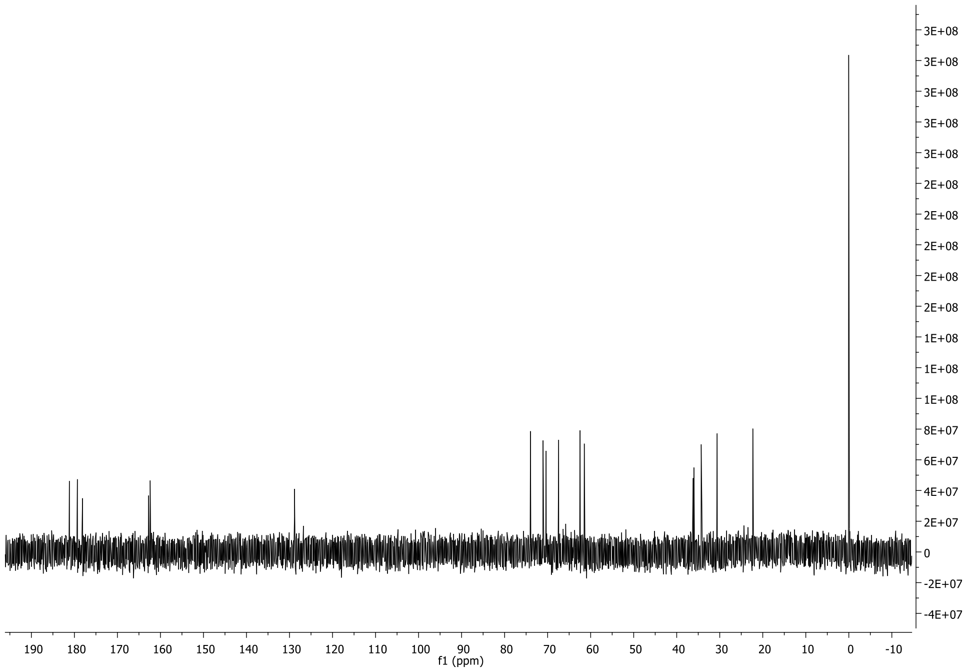

Figure S12. ^{13}C NMR spectrum of bostrychine B in D_2O at 150 MHz

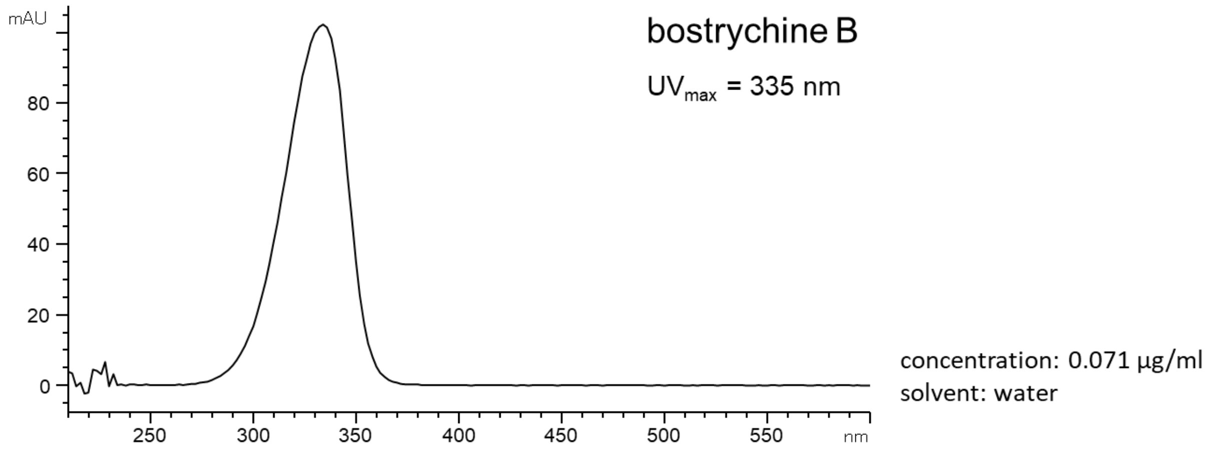

Figure S13.UV spectrum of bostrychine B

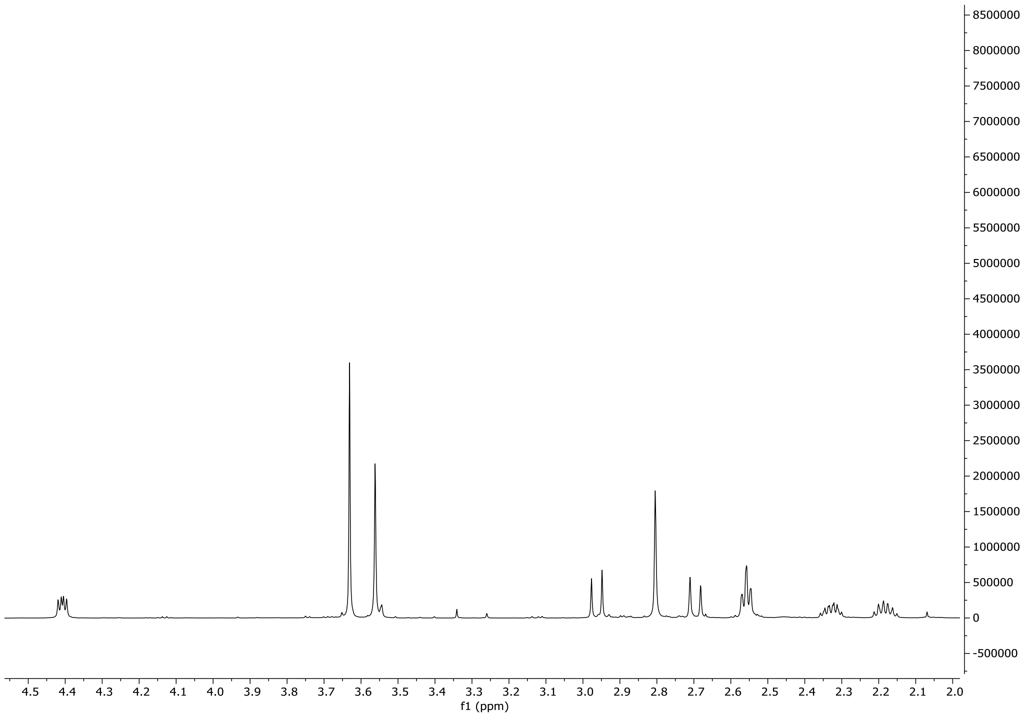

Figure S14.^{1}H NMR spectrum of bostrychine C in D_2O at 600 MHz

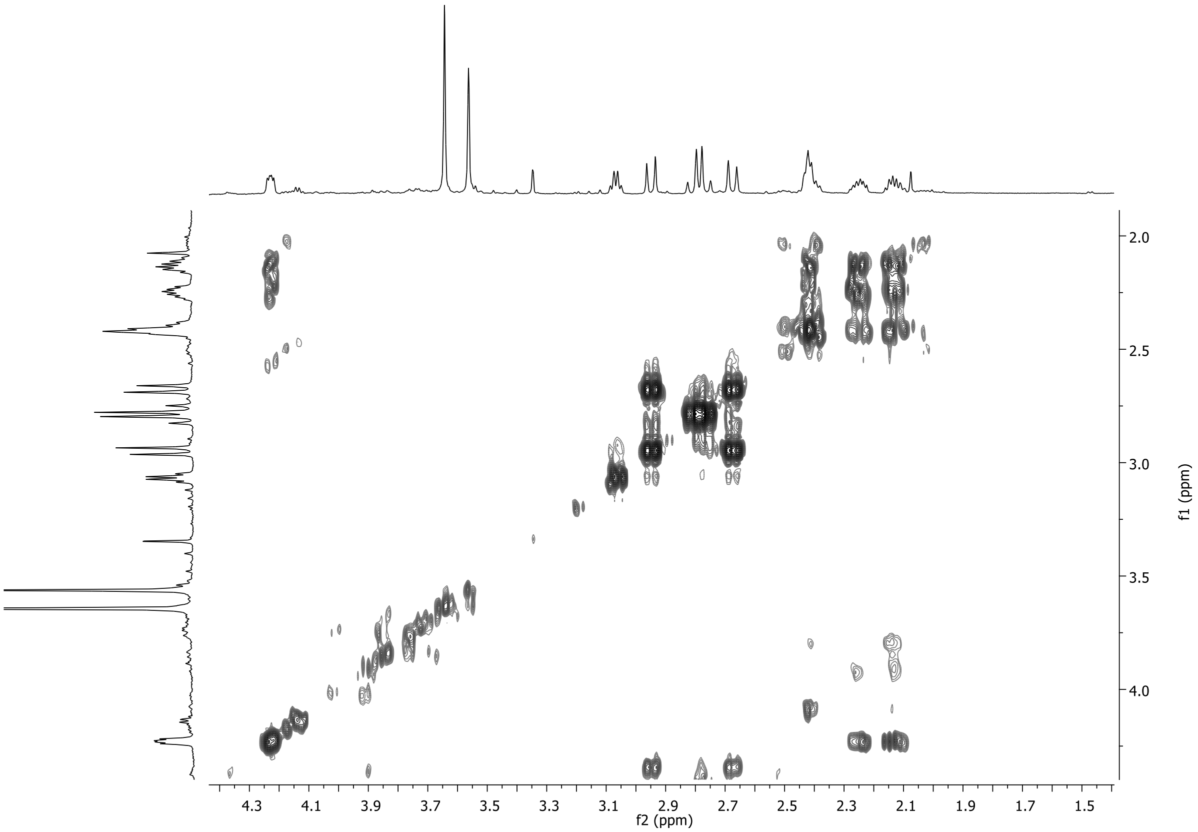

Figure S15. COSY spectrum of bostrychine C in D_2O at 600 MHz

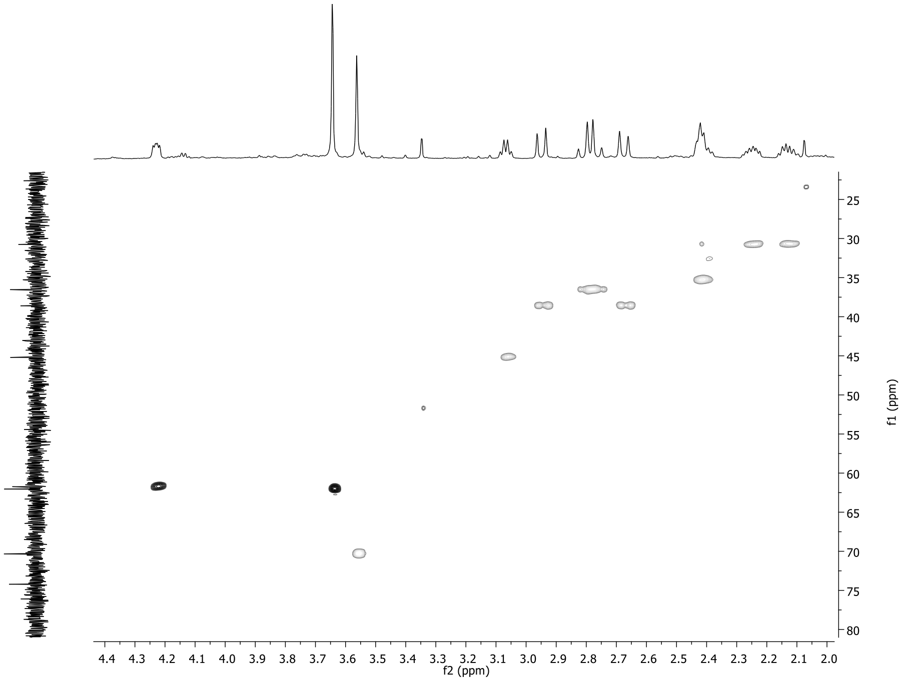

Figure S16. HSQC spectrum of bostrychine C in D_2O at 600 MHz

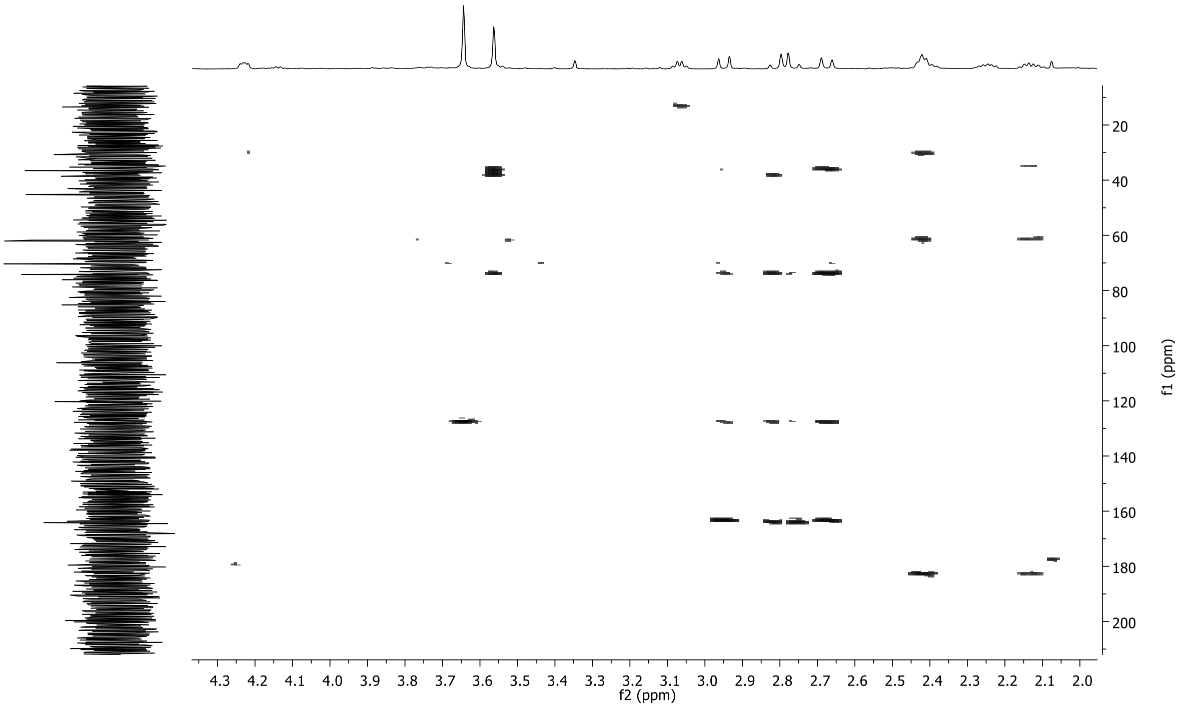

Figure S17. HMBC spectrum of bostrychine C in D_2O at 600 MHz

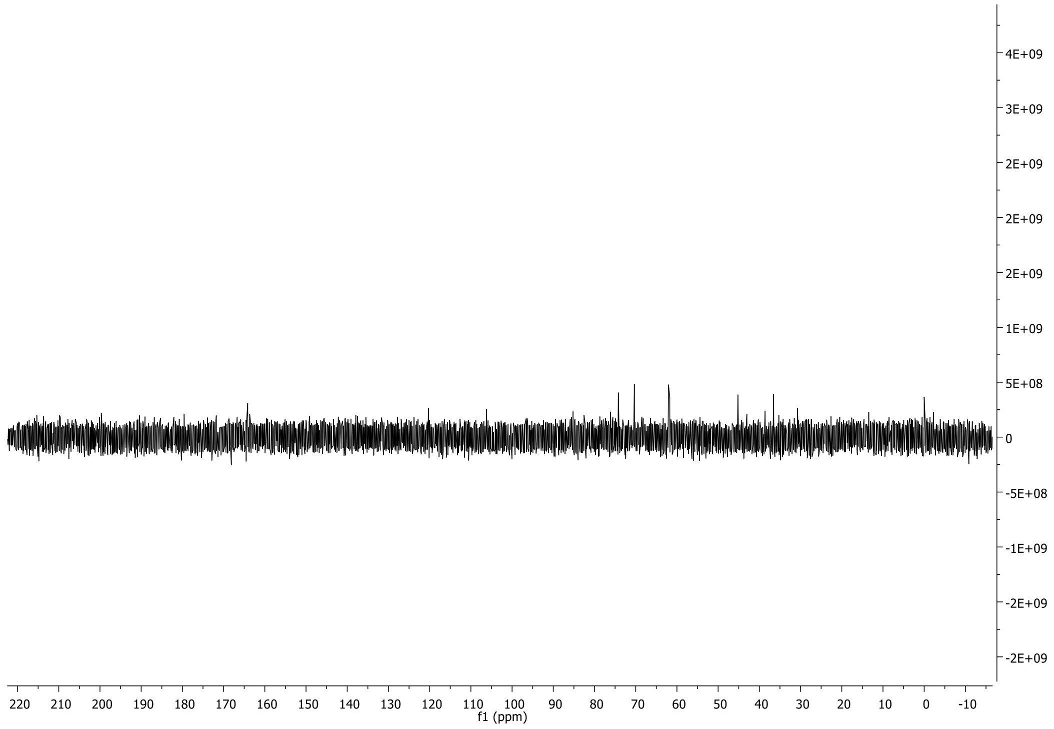

Figure S18.^{13}C NMR spectrum of bostrychine C in D_2O at 150 MHz

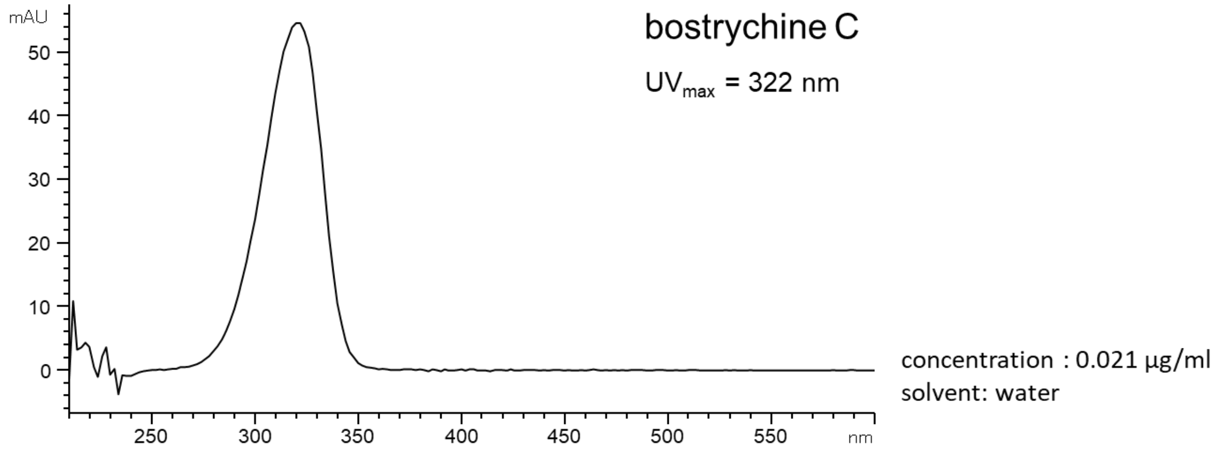

Figure S19.UV spectrum of bostrychine C

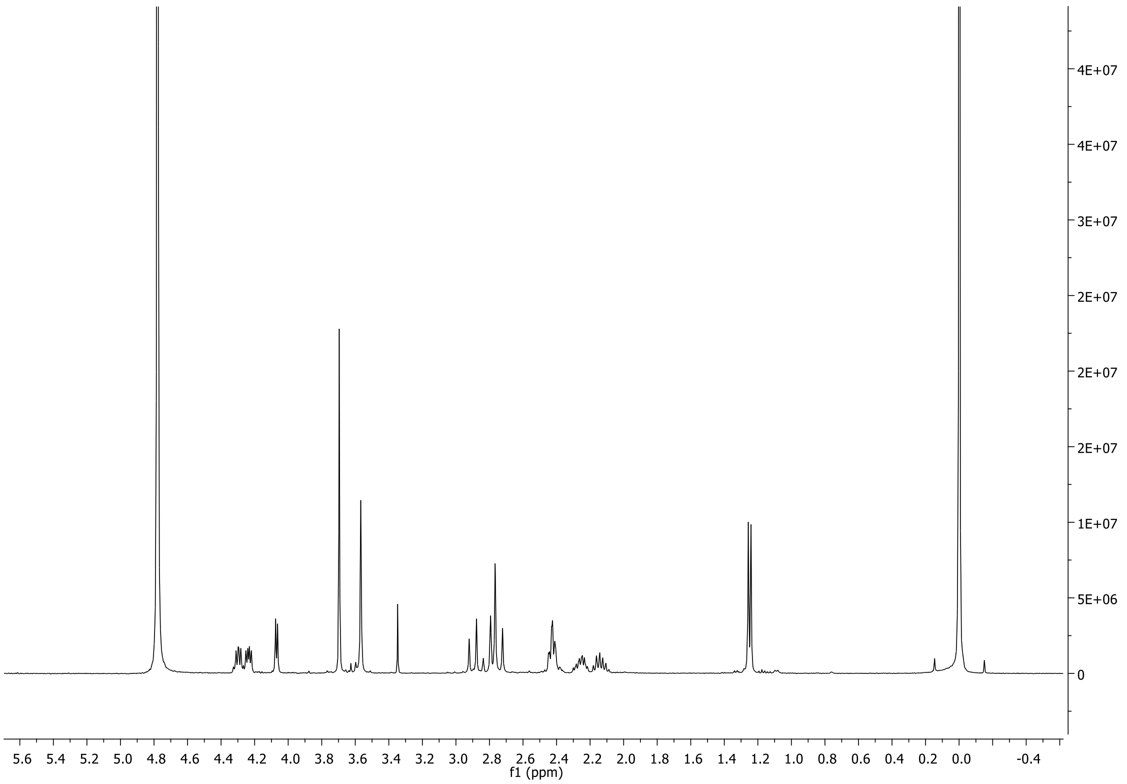

Figure S20.^{1}H NMR spectrum of bostrychine D in D_2O at 600 MHz

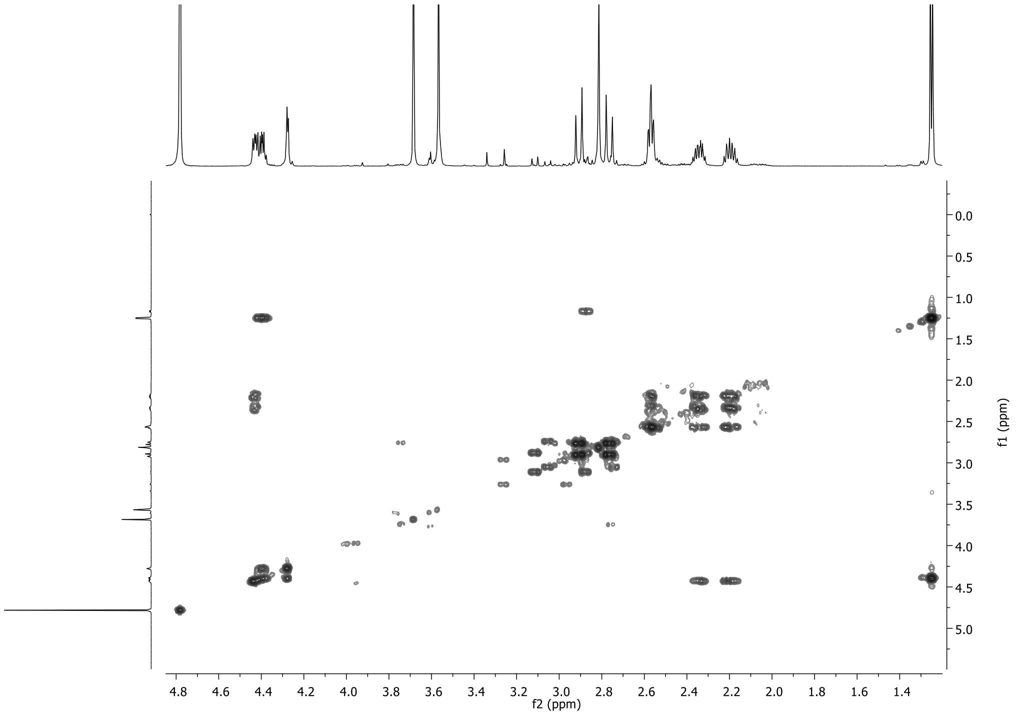

Figure S21. COSY spectrum of bostrychine D in D_2O at 600 MHz

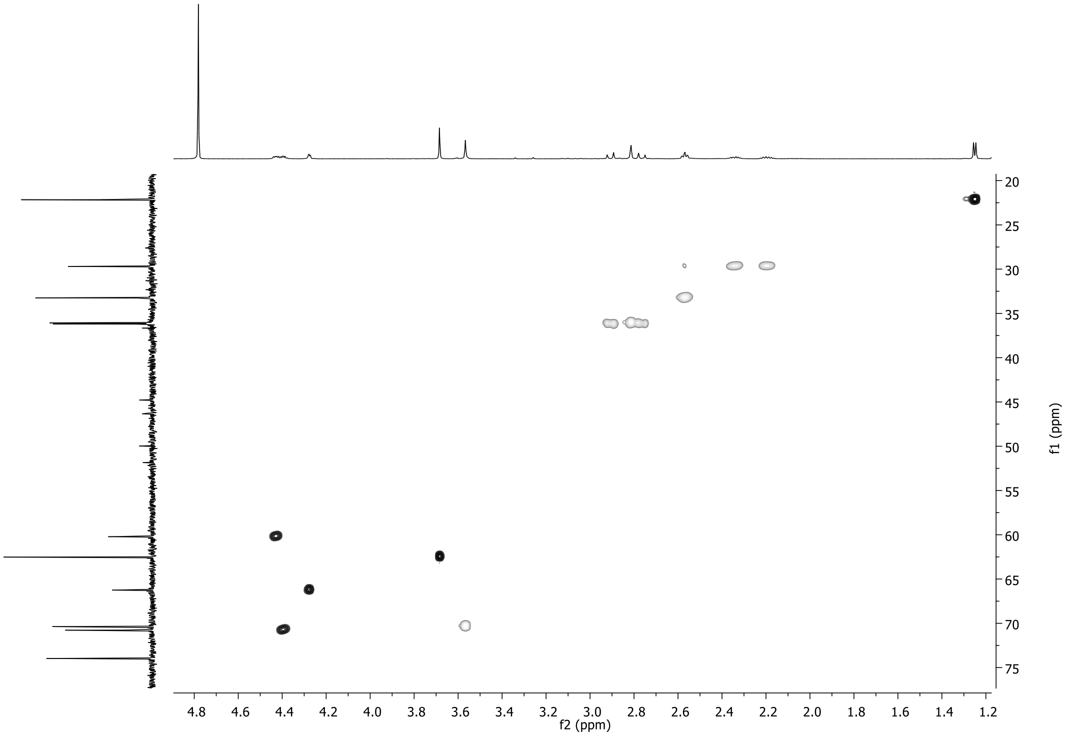

Figure S22. HSQC spectrum of bostrychine D in D_2O at 600 MHz

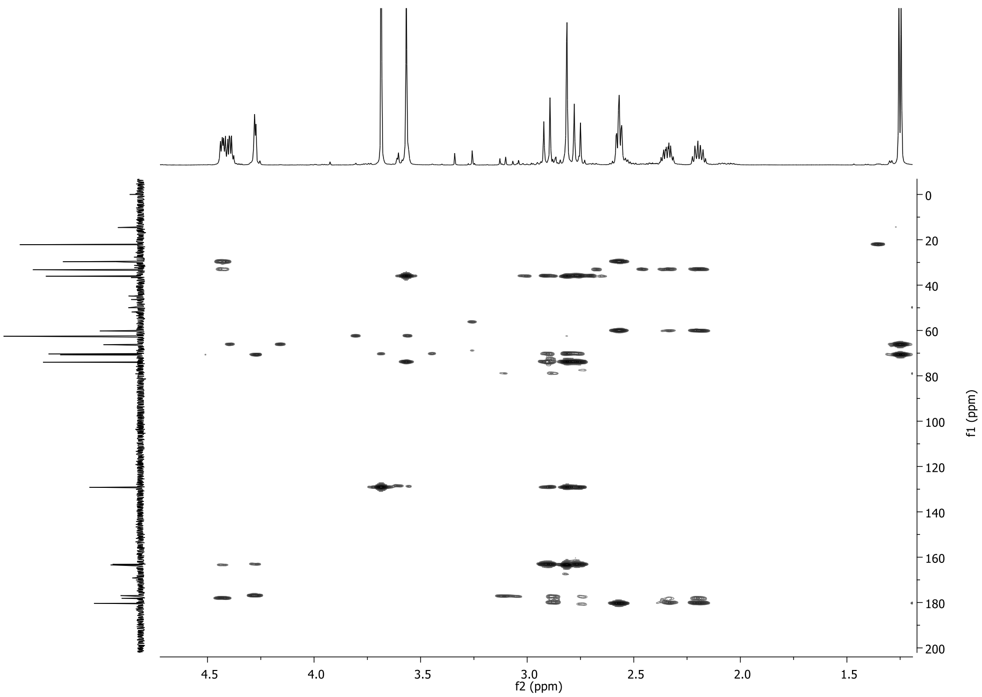

Figure S23. HMBC spectrum of bostrychine D in D_2O at 600 MHz

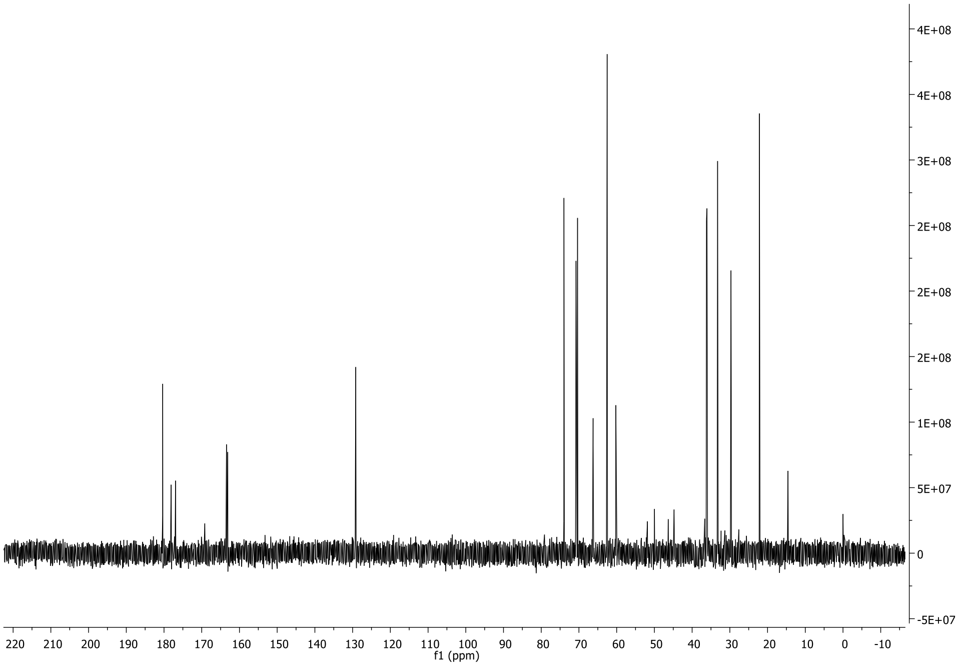

Figure S24. ^{13}C NMR spectrum of bostrychine D in D_2O at 150 MHz

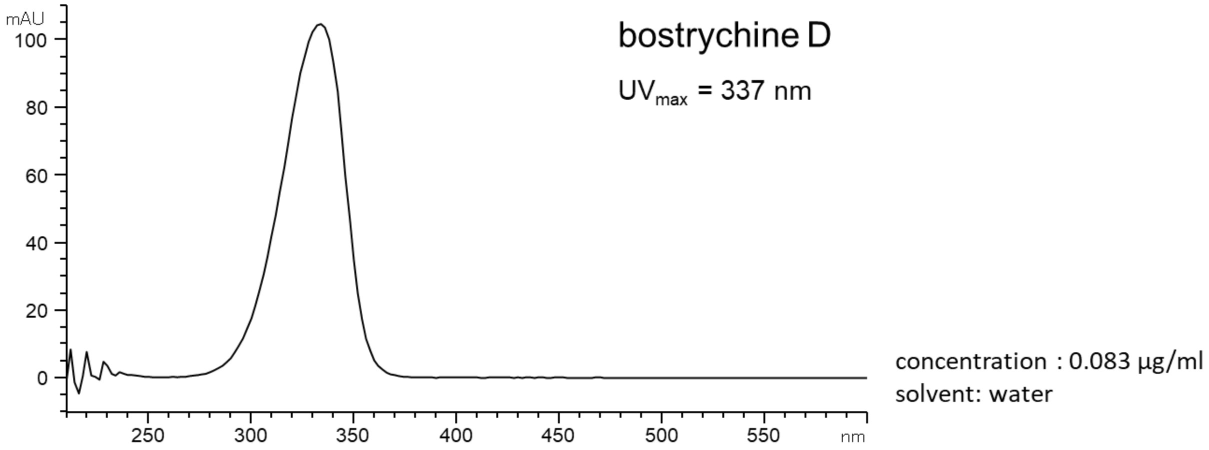

Figure S25.UV spectrum of bostrychine D

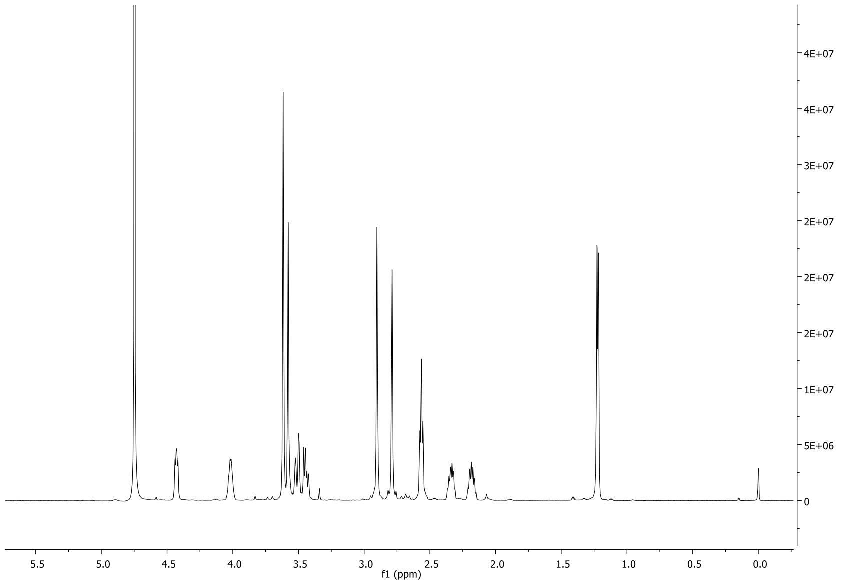

Figure S26.^{1}H NMR spectrum of bostrychine E in D_2O at 600 MHz

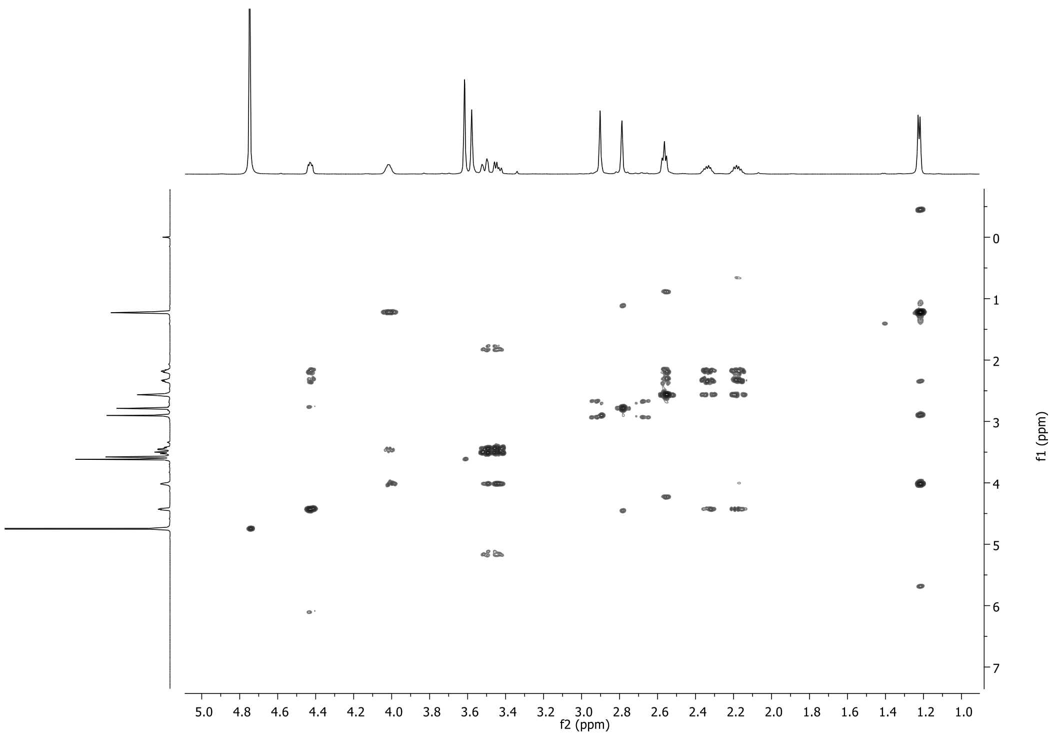

Figure S27. COSY spectrum of bostrychine E in D_2O at 600 MHz

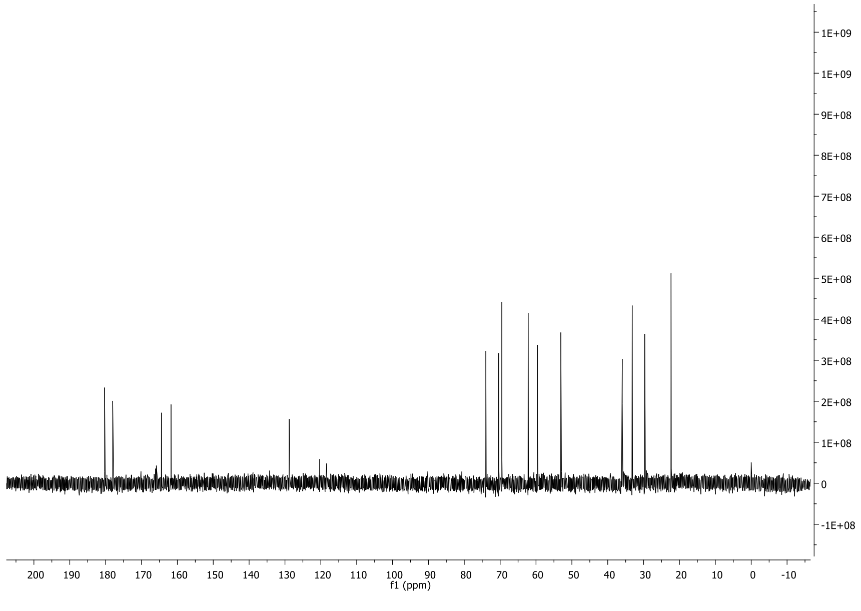

Figure S28. ^{13}C NMR spectrum of bostrychine E in D_2O at 150 MHz

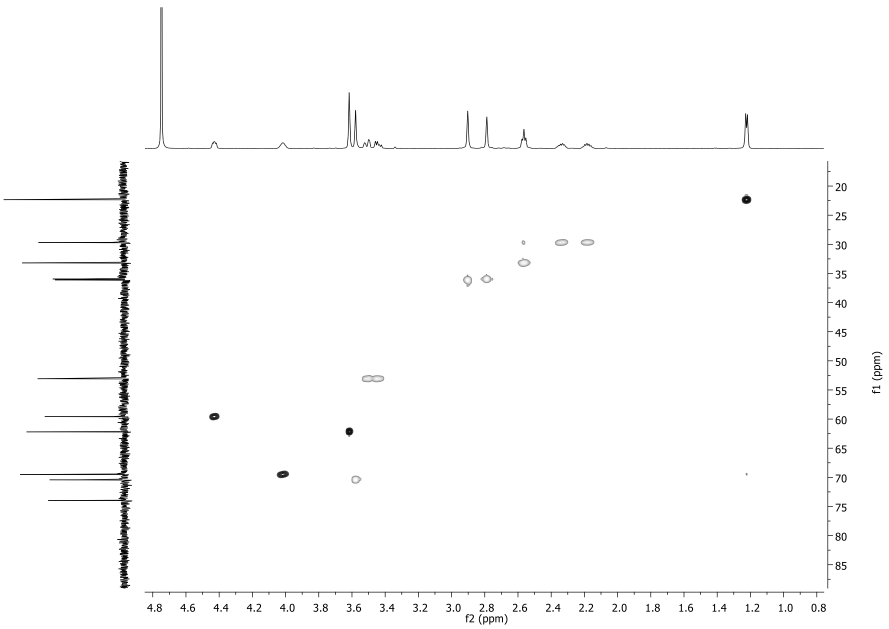

Figure S29. HSQC spectrum of bostrychine E in D_2O at 600 MHz

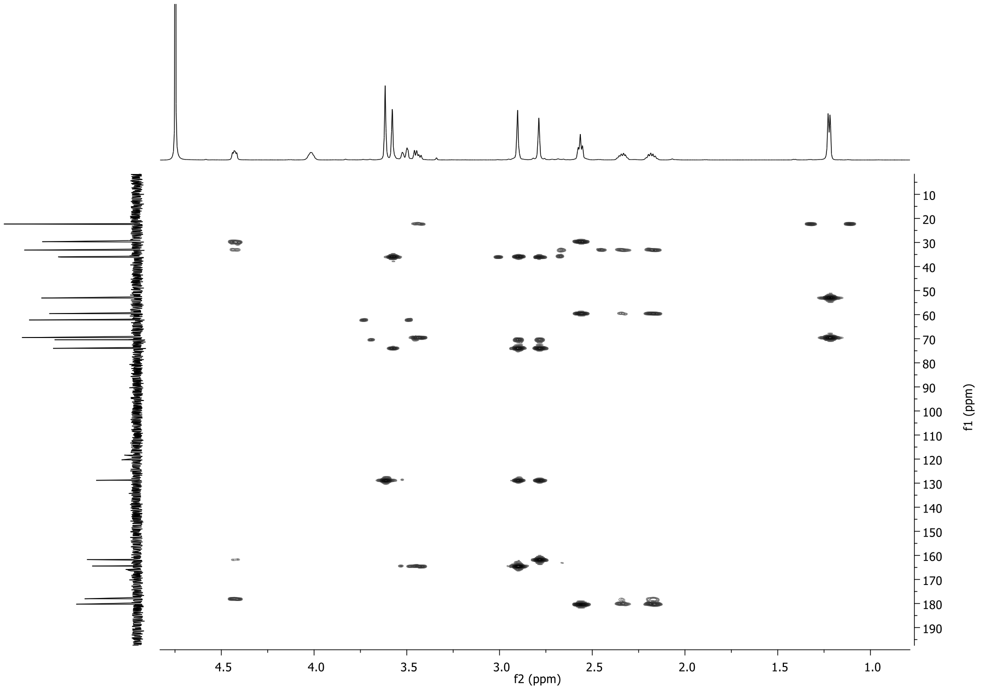

Figure S30. HMBC spectrum of bostrychine E in D_2O at 600 MHz

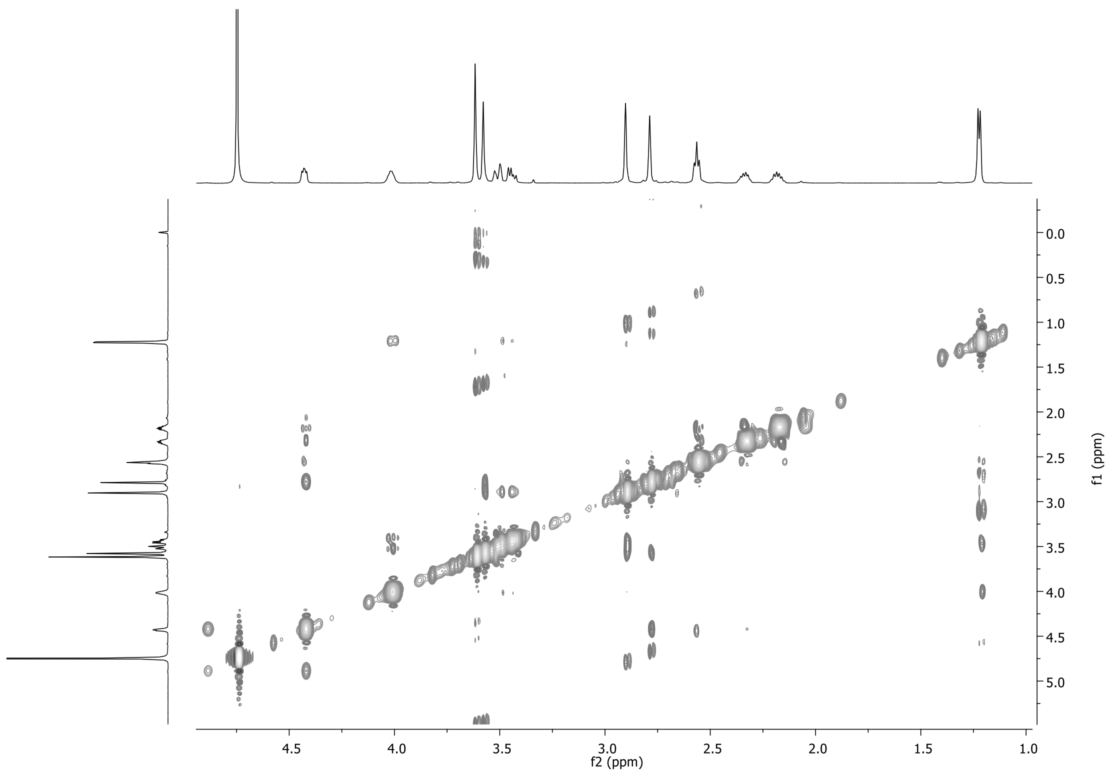

Figure S31. NOESY spectrum of bostrychine E in D_2O at 600 MHz

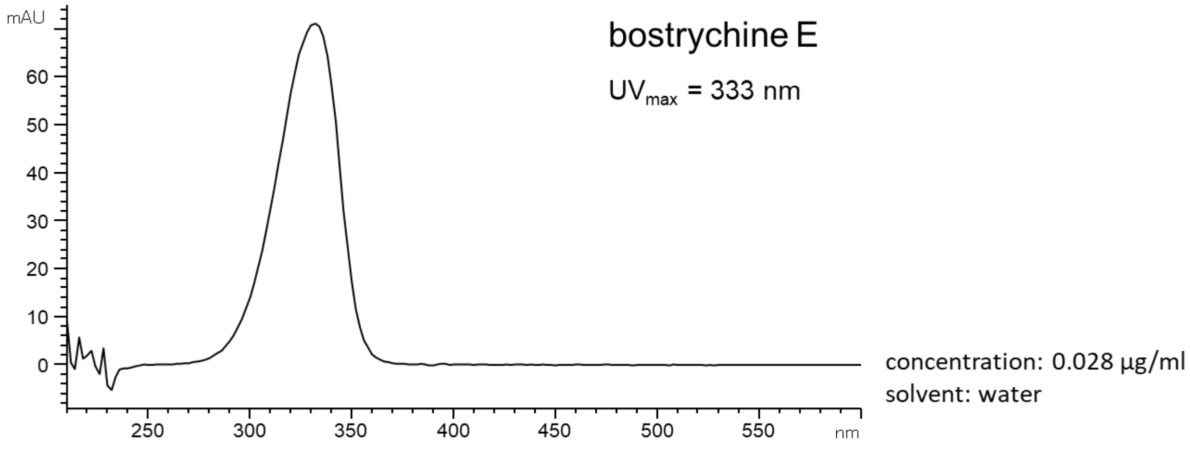

Figure S32.UV spectrum of bostrychine E

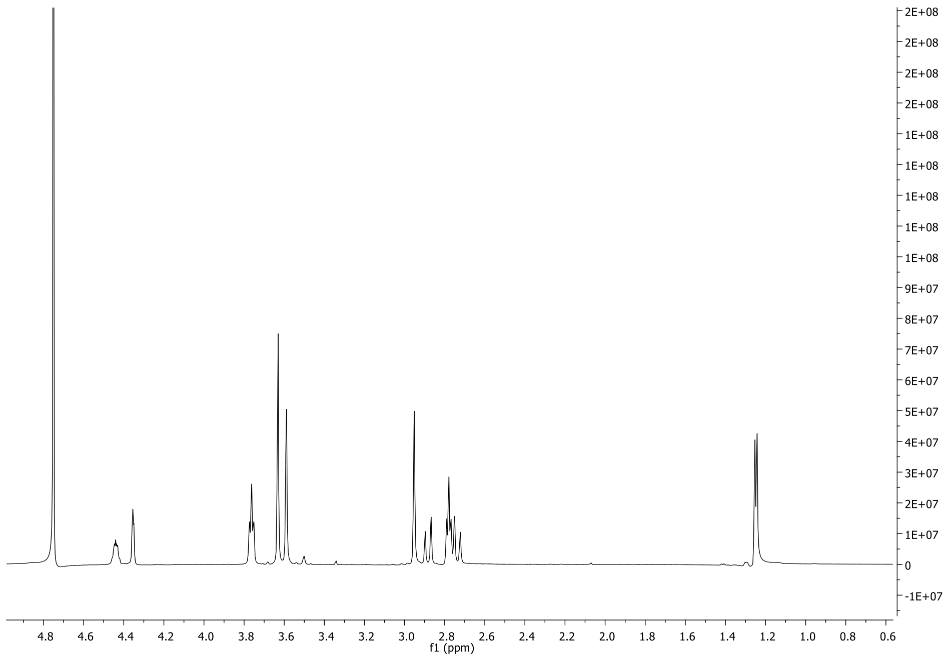

Figure S33.^{1}H NMR spectrum of bostrychine F in D_2O at 600 MHz

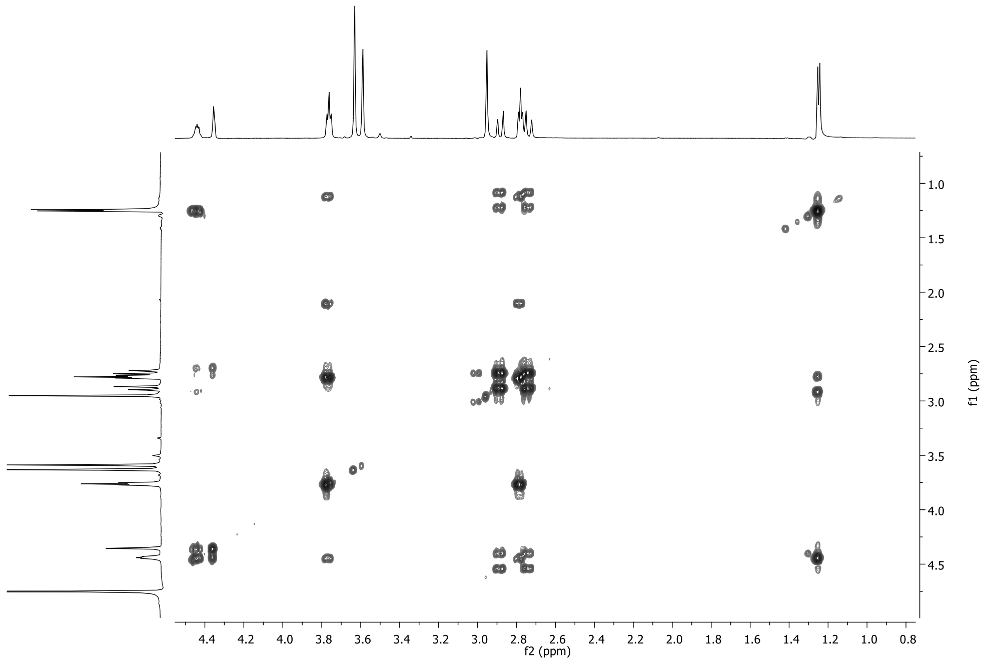

Figure S34. COSY spectrum of bostrychine F in D_2O at 600 MHz

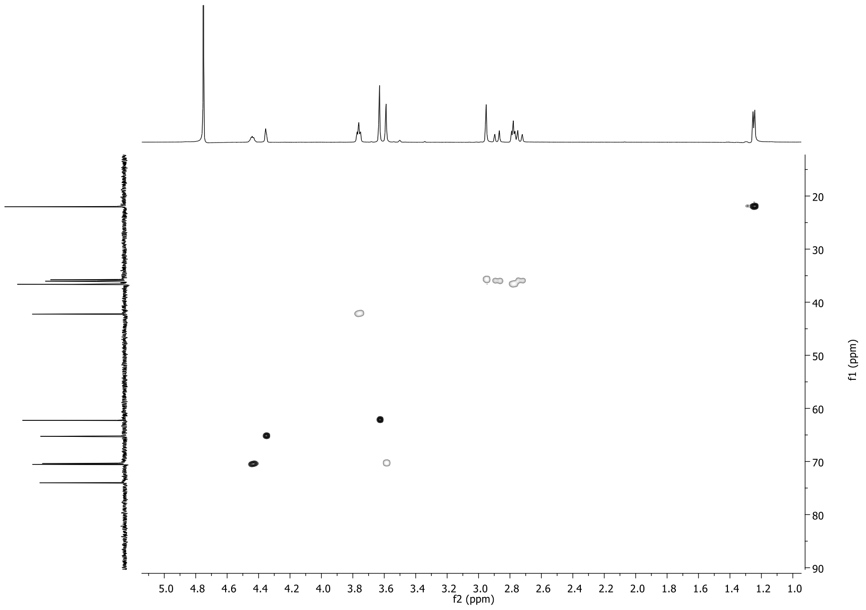

Figure S35. HSQCspectrum of bostrychine F in D_2O at 600 MHz

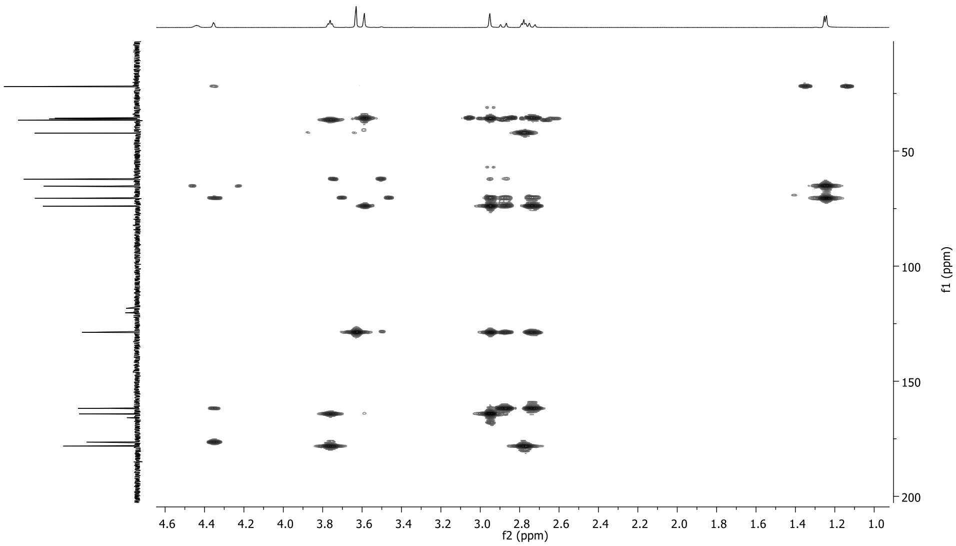

Figure S36. HMBC spectrum of bostrychine F in D_2O at 600 MHz

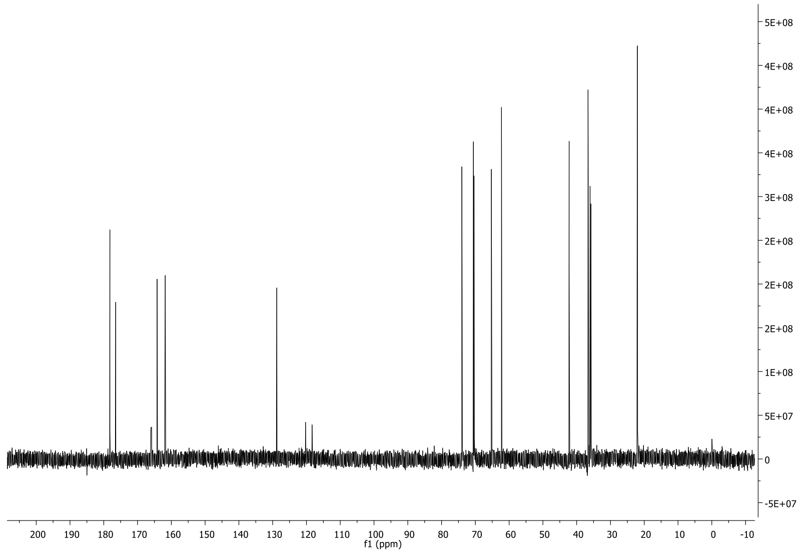

Figure S37 ^{13}C NMR spectrum of bostrychine F in D_2O at 150 MHz

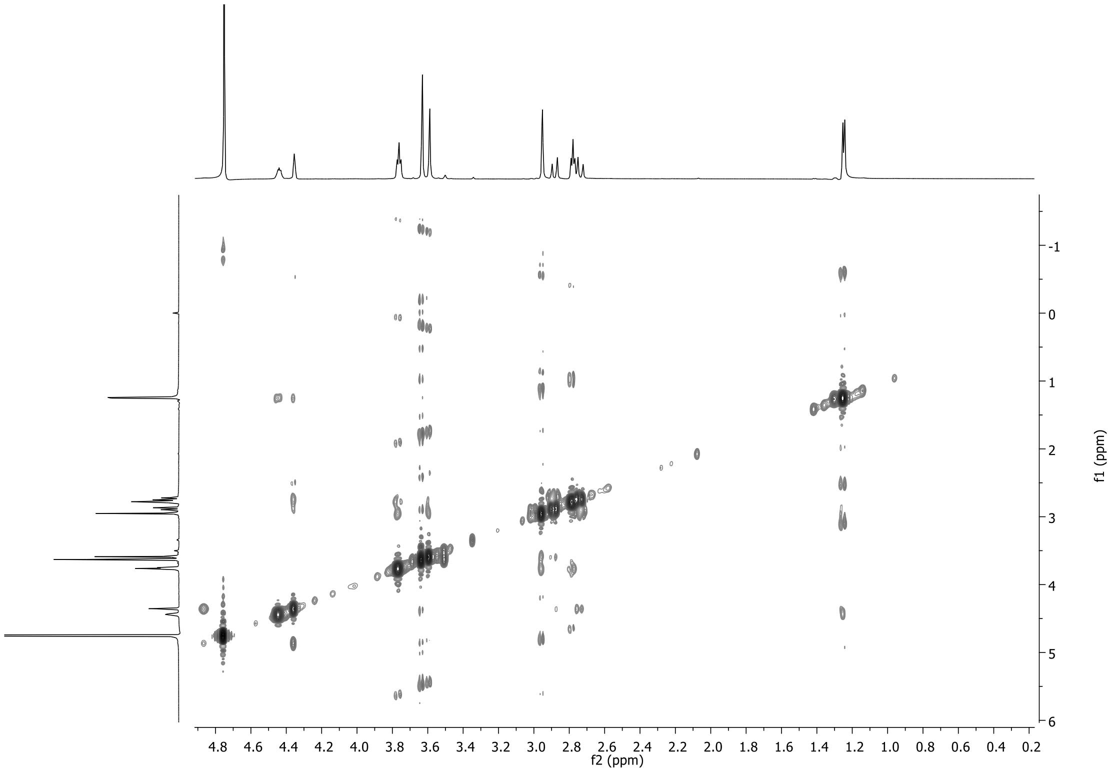

Figure S38. NOESY spectrum of bostrychine F in D_2O at 600 MHz

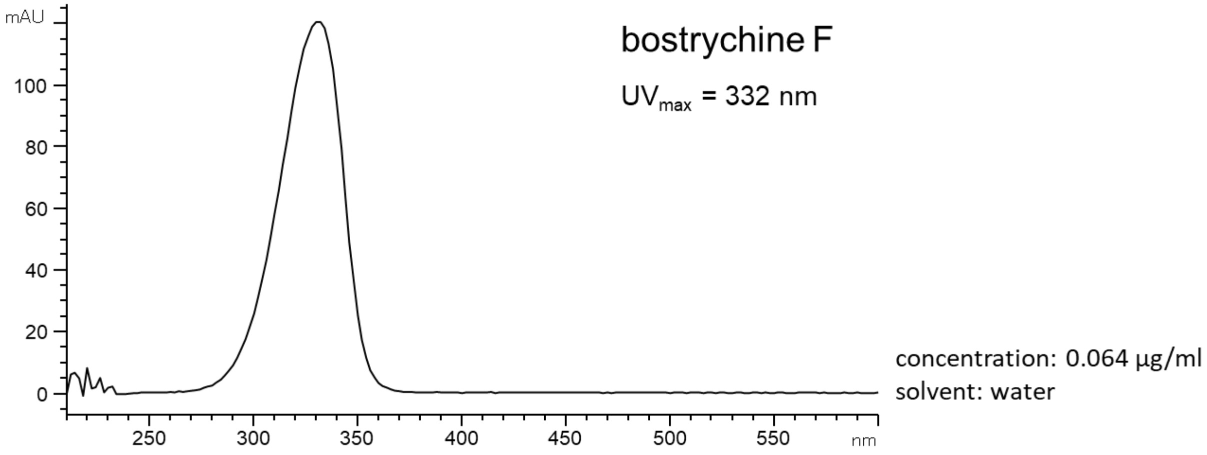

Figure S39.UV spectrum of bostrychine F
